# Iron-Based Nanoparticles as Delivery Tools

**DOI:** 10.3390/ph19050654

**Published:** 2026-04-22

**Authors:** Keykavous Parang, Rajesh Vadlapatla, Ajoy Koomer, Victoria Moran, Lanie Jackson, Amir Nasrolahi Shirazi

**Affiliations:** 1 Center for Targeted Drug Delivery, Department of Biomedical and Pharmaceutical Sciences, Chapman University School of Pharmacy, Harry and Diane Rinker Health Science Campus, 9401 Jeronimo Rd., Irvine, CA 92618, USA; parang@chapman.edu; 2 Department of Pharmaceutical Sciences, College of Pharmacy, Marshall B. Ketchum University, 2575 Yorba Linda Blvd., Fullerton, CA 92831, USA; rvadlapatla@ketchum.edu (R.V.); akoomer@ketchum.edu (A.K.); victoriamoran.cop28@ketchum.edu (V.M.); laniejackson.cop28@ketchum.edu (L.J.)

**Keywords:** drug delivery, Fe_3_O_4_, γ-Fe_2_O_3_, Fe_3_O_4_@SiO_2_, iron oxide nanoparticles, magnetic nanoparticles, SPIONs

## Abstract

Iron-based nanoparticles, particularly iron oxide nanostructures (IONPs), have emerged as versatile and clinically relevant platforms for drug delivery and theranostic applications. Among these, superparamagnetic iron oxide nanoparticles (SPIONs), including magnetite (Fe_3_O_4_) and maghemite (γ-Fe_2_O_3_), are the most extensively investigated due to their biocompatibility, magnetic responsiveness, and established safety profiles. Their unique superparamagnetic behavior enables external magnetic-field-guided targeting, magnetic resonance imaging (MRI) contrast enhancement, and magnetically triggered hyperthermia, enabling simultaneous diagnosis and therapy. Surface functionalization with polymers, silica, lipids, peptides, and biomolecules further improves colloidal stability, circulation time, targeting specificity, and controlled drug release. Core–shell architectures and multifunctional hybrid systems have expanded the therapeutic scope of iron nanoparticles, integrating chemotherapy, gene delivery, photothermal therapy, and Fenton reaction–mediated catalytic therapy. Despite promising preclinical outcomes, challenges remain regarding long-term biosafety, oxidative stress induction, biodistribution, large-scale reproducibility, and regulatory translation. This review summarizes the physicochemical properties, synthesis strategies, surface-engineering approaches, drug-loading mechanisms, and biomedical applications of iron-based nanoparticles, highlighting recent advances in multifunctional and peptide-functionalized systems. Critical considerations for clinical translation and future perspectives in precision nanomedicine are also discussed.

## 1. Introduction

Drug delivery refers to the formulation, technologies, and systems used to transport a therapeutic agent to its intended site of action in the body with the appropriate dose, timing, and duration to achieve a desired pharmacological effect while minimizing toxicity to healthy tissues [1]. Conventional drug administration, via oral, intravenous, or intramuscular routes, faces fundamental limitations that constrain therapeutic efficacy across a broad range of diseases, most critically in oncology. Systemically administered drugs distribute non-selectively throughout the body, resulting in peak plasma concentrations that cause dose-limiting toxicity to healthy organs while simultaneously failing to sustain therapeutic concentrations at the tumor site. Poor aqueous solubility, rapid enzymatic degradation, short circulation half-lives, and multidrug resistance mechanisms further erode the clinical effectiveness of many potent therapeutic compounds [2,3]. These limitations have driven decades of research into advanced drug-delivery systems designed to overcome the barriers of conventional pharmacotherapy.

### 1.1. Metal Nanoparticles in Drug Delivery

Metal-based nanoparticles (MNPs) have emerged as a pivotal class of nanomaterials owing to their distinctive physicochemical properties arising at the nanoscale. Their tunable dimensions, high surface-to-volume ratio, and adaptable surface chemistry enable extensive functional modification, allowing simultaneous therapeutic and diagnostic (theranostic) applications [4]. These attributes include targeted delivery capability, imaging compatibility, and controlled drug release, positioning MNPs as superior platforms compared with many conventional drug delivery systems [5].

A wide spectrum of MNPs has been investigated, such as gold nanoparticles (AuNPs), silver nanoparticles (AgNPs), iron oxide nanoparticles (Fe_3_O_4_ NPs, IONPs), platinum nanoparticles (PtNPs), zinc oxide nanoparticles (ZnO NPs), selenium nanoparticles (SeNPs), and gadolinium nanoparticles (GdNPs). These nanostructures can be fabricated via chemical, physical, or biologically mediated synthesis methods and have been widely explored for both therapeutic and diagnostic applications [6].

Among the broad spectrum of metal-based nanoparticles explored for biomedical applications, iron nanoparticles have emerged as a uniquely compelling platform, distinguished by a combination of properties. Although MNPs encompass diverse functionalities tailored to specific biomedical needs, iron nanoparticles have recently gained significant attention for their magnetic responsiveness, biocompatibility, and broad therapeutic potential [7]. Their inherent magnetic responsiveness enables active, externally guided targeting, a capability that gold, silver, platinum, or zinc-based nanoparticles fundamentally lack. Critically, iron is an endogenous element with well-established metabolic pathways, allowing iron nanoparticles to be safely processed by the body. Compared with other MNPs such as manganese, copper, or cobalt, iron nanoparticles exhibit superior biocompatibility and more favorable bioaccumulation profiles, supporting their safer use in biomedical applications [8,9,10]. As a result, iron nanoparticles are considered suitable for diagnostic and therapeutic purposes while presenting a lower risk of toxicity [11,12]. This convergence of magnetic functionality, biological compatibility, and metabolic integration makes iron nanoparticles not merely an alternative to other MNP platforms but a distinctly superior choice for simultaneous diagnostic and therapeutic applications.

Among MNPs, iron nanoparticles exhibit superparamagnetic properties, enabling magnetic targeting and their use as contrast agents in MRI [13]. Iron and GdNPs represent two major classes of magnetic nanomaterials used in biomedical imaging; however, they differ fundamentally in their magnetic behavior, MRI contrast mechanisms, and therapeutic potential. Iron Oxide Nanoparticles (IONPs) display superparamagnetism, meaning they strongly respond to external magnetic fields without retaining residual magnetization. As a result, they primarily function as T2 (negative) MRI contrast agents, producing signal darkening in images. Their strong magnetic responsiveness also enables additional therapeutic applications, including magnetic hyperthermia, magnetically guided drug delivery, and cell tracking [14,15]. In contrast, GdNPs are paramagnetic and primarily act as T1 (positive) MRI contrast agents that enhance signal intensity and produce brighter images. Unlike IONPs, GdNPs do not exhibit strong magnetic responsiveness and therefore are generally not suitable for magnetic targeting or hyperthermia-based therapies. Instead, their primary advantage lies in their high longitudinal relaxivity, which provides superior image contrast at lower concentrations. Additionally, IONPs are typically considered more suitable for long-term biomedical applications due to their biodegradability into endogenous iron pathways, whereas gadolinium-based materials require careful design and stabilization because the release of free gadolinium ions may lead to toxicity concerns [16,17,18,19,20,21].

To further contextualize the value of iron nanoparticles within the broader nanocarrier landscape, it is instructive to compare them with the major competing drug delivery platforms. Liposomes, the most clinically advanced nanocarrier class, with multiple FDA-approved formulations including Doxil^®^ [22], offer high biocompatibility and excellent encapsulation of both hydrophilic and hydrophobic drugs, but lack intrinsic imaging capability, cannot be magnetically guided, and are susceptible to lipid oxidation and cargo leakage during circulation. Polymeric nanoparticles (e.g., PLGA) [23] provide tunable degradation rates and sustained release profiles, but like liposomes, lack magnetic responsiveness and are limited in theranostic functionality. AuNPs offer unique photothermal properties and straightforward surface chemistry, but their non-biodegradability raises concerns about long-term tissue accumulation, and they offer no inherent drug-loading advantage over IONPs [24]. Dendrimers enable precise molecular architecture and high surface ligand density but suffer from complex synthesis, batch variability, and potential cationic cytotoxicity at higher generations [25]. In this context, IONPs occupy a distinctive position: they are the only clinically relevant nanocarrier class that combines active magnetic guidance, intrinsic MRI contrast, biodegradability through endogenous iron pathways, and versatile surface functionalization within a single, scalable platform [26]. Their primary limitations relative to liposomes and polymeric carriers are lower drug loading capacity for hydrophilic molecules and greater sensitivity to aggregation, limitations that core-shell architectures and stimuli-responsive coatings are increasingly designed to address. A comprehensive understanding of these comparative trade-offs is essential for rational selection of nanocarrier type for specific clinical indications and should inform the design of next-generation IONP platforms [27,28].

When IONPs are coated with polymers, peptides, or other biocompatible materials, they can deliver drugs selectively to tumors or inflamed tissues under the guidance of an external magnetic field [29]. Their favorable biocompatibility and biodegradability support their application as multifunctional theranostic systems. Additionally, they have demonstrated enhanced cellular internalization and controlled release profiles for anticancer agents such as 5-fluorouracil (5-FU) [30].

Iron nanoparticles act as pH-responsive carriers that degrade preferentially in acidic tumor microenvironments, triggering localized drug release and reactive oxygen species (ROS)-mediated cytotoxicity. This intrinsic responsiveness underscores their promise in targeted cancer therapy [31].

Furthermore, their ability to participate in redox reactions enables applications in cancer therapy through oxidative stress modulation and ROS generation, which can promote cancer cell death [7,8]. Surface modification with targeting ligands, polymers, or bioactive molecules further enhances nanoparticle stability, prolongs circulation time, and improves targeting efficiency and therapeutic performance [17].

Our group has previously reported several functionalized MNP systems, including gold, selenium, and gadolinium-based nanoplatforms, as multifunctional constructs capable of advancing drug delivery and diagnostic technologies [32,33,34,35,36,37]. This review provides a comprehensive overview of the significance of iron in the human body and elaborates on the applications of iron nanoparticles for drug delivery, current limitations, and future research directions.

While numerous reviews have examined magnetic nanoparticles broadly or focused narrowly on SPIONs as MRI contrast agents [38,39,40,41,42], the present review is distinguished by several features. First, it systematically covers three mechanistically distinct classes of iron-based nanoparticles, IONPs/SPIONs, zero-valent iron nanoparticles (nZVI), and iron-based core–shell architectures, within a unified drug delivery framework, an approach not consistently adopted in prior reviews. Second, we provide an integrated discussion of iron physiology, including systemic iron metabolism, ferroptosis, immune modulation, and neurocognitive roles, as a biological foundation for understanding both the therapeutic rationale and the safety considerations of iron nanoparticles in vivo. Third, we specifically highlight peptide-functionalized iron nanoparticle systems and Fenton reaction-mediated catalytic therapy as emerging therapeutic modalities. Finally, we discuss the integration of artificial intelligence (AI) in the rational design of iron nanoparticle drug delivery systems, reflecting the most recent directions in the field.

### 1.2. Iron

Iron (Fe) is an essential trace element required for normal growth, cellular respiration, oxygen transport, and systemic metabolic homeostasis [43,44]. As one of the most abundant transition metals in the human body, iron participates in diverse biochemical processes critical for survival. In humans, iron exists primarily in two biologically relevant oxidation states, ferrous (Fe^2+^) and ferric (Fe^3+^), allowing it to participate in reversible redox reactions essential for energy production and enzymatic activity [44,45].

**Figure 1:**
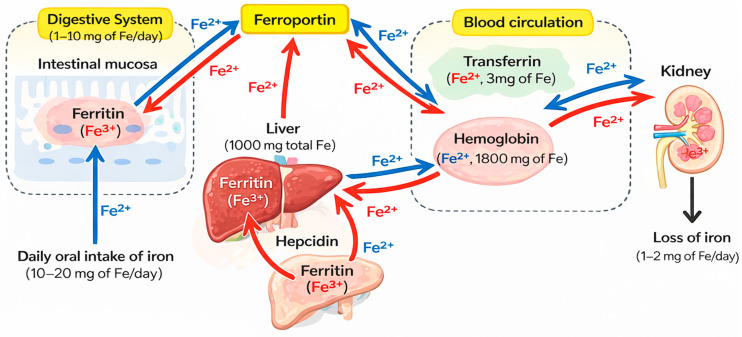
Iron distribution in the human body and the key organs responsible for regulating iron metabolism, adapted from [48] under the Creative Commons CC BY 4.0 license. The Figure was reproduced with permission from [48]—[Abe, C.; Miyazawa, T.; Miyazawa, T]. [Current Use of Fenton Reaction in Drugs and Food], published by [MDPI], [2022].

Dietary iron exists in two principal forms: heme iron, derived from animal sources, and non-heme iron, found in plant-based foods and fortified products. Heme iron, which is present in hemoglobin, myoglobin, and cytochromes, contains an iron atom coordinated within a porphyrin (heme) ring and is typically in the ferrous (Fe^2+^) state in hemoglobin and myoglobin, enabling oxygen binding; oxidation to the ferric (Fe^3+^) state results in methemoglobin, which cannot effectively bind oxygen. This form of iron is efficiently absorbed in the duodenum via specific heme transport mechanisms. In contrast, non-heme iron is not bound to a heme ring and is commonly present as ferric (Fe^3+^) iron in foods and the intestinal lumen; prior to absorption, it must be reduced to the ferrous (Fe^2+^) form by intestinal reductases to allow uptake through divalent metal transporter 1 (DMT1) [46]. Once absorbed, iron can be stored intracellularly in ferritin, utilized in metabolic processes, or exported via ferroportin, the only known cellular iron exporter, before binding to transferrin for systemic distribution [47]. Figure 1 shows iron distribution in the human body and the key organs responsible for regulating iron metabolism.

Systemic iron homeostasis is tightly regulated by hepcidin, a liver-derived peptide hormone that controls ferroportin expression and prevents excessive iron accumulation [49]. Because the human body lacks a regulated pathway for iron excretion, iron balance depends primarily on controlled intestinal absorption and macrophage-mediated recycling of senescent erythrocytes [50,51].

Iron is incorporated into numerous metalloproteins and enzymes mediating oxygen transport, mitochondrial respiration, DNA synthesis, and cellular proliferation [50,51]. Hemoglobin and myoglobin rely on iron for oxygen binding and delivery, while iron–sulfur cluster–containing proteins and cytochromes are essential components of the electron transport chain [50,51]. Consequently, iron deficiency impairs oxidative phosphorylation, reduces ATP production, and compromises tissue oxygenation, resulting in fatigue and diminished physical performance [43,52].

#### 1.2.1. Iron in Oxygen Transport and Energy Metabolism

Approximately two-thirds of total body iron is incorporated into hemoglobin, facilitating the transport of oxygen from the lungs to peripheral tissues [43]. Iron is also present in myoglobin, supporting oxygen storage in muscle tissue [45].

Beyond oxygen transport, iron is fundamental to mitochondrial ATP generation. Iron-sulfur clusters and heme-containing cytochromes enable electron transfer reactions within complexes I–IV of the respiratory chain [53]. Disruption of iron availability impairs mitochondrial function and energy metabolism, contributing to the clinical manifestations of iron deficiency anemia [52].

However, excessive iron accumulation promotes oxidative stress via Fenton chemistry, in which Fe^2+^ catalyzes the formation of hydroxyl radicals from hydrogen peroxide [44,48]. This redox activity emphasizes the importance of tightly controlled regulatory systems that maintain iron homeostasis.

Although Fenton hypothesized that H_2_O_2_ and iron (II) drive oxidation, some researchers questioned whether this one-electron reduction process produces hydroxyl radicals. The Haber–Weiss reaction describes the iron-catalyzed decomposition of hydrogen peroxide (H_2_O_2_) to generate reactive oxygen species, particularly hydroxyl radicals (OH•) [54,55]. While Fenton initially proposed H_2_O_2_ oxidation by Fe^2+^, the direct formation of OH• was debated [56]. Haber and Wilstätter suggested that OH• participates in radical chain reactions initiated by catalase, and later, Haber and Weiss demonstrated that Fe^2+^ reacts with H_2_O_2_ to produce OH•, which propagates a chain reaction with superoxide (O_2_•^−^) before termination by Fe^2+^ oxidation [55]. The reaction outcome is influenced by pH and iron concentration, yielding oxo-iron (IV) species at neutral pH and OH• at acidic conditions [57,58,59,60].

**Figure 2:**
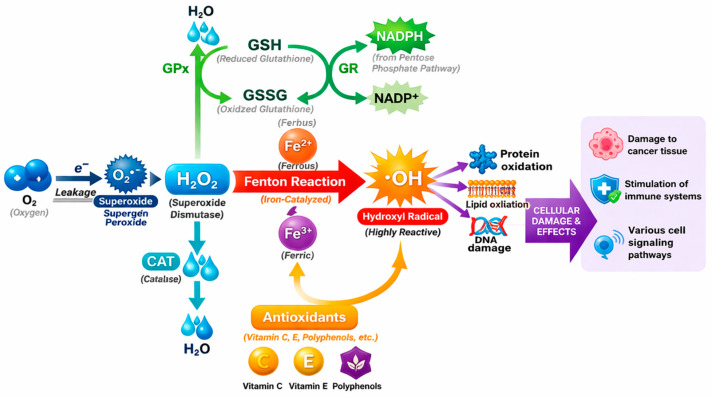
ROS formation through the Fenton reaction in biological systems. CAT, catalase; GPx, glutathione peroxidase; GR, glutathione reductase; GSH, reduced glutathione; GSSG, oxidized glutathione; NADPH, reduced nicotinamide adenine dinucleotide phosphate; NADP^+^, oxidized nicotinamide adenine dinucleotide phosphate; adapted from [48] under the Creative Commons CC BY 4.0 license [48]. The Figure was reproduced with permission from [48]—[Abe, C.; Miyazawa, T.; Miyazawa, T]. [Current Use of Fenton Reaction in Drugs and Food], published by [MDPI], [2022].

Two mechanisms, the radical and the complex, account for product diversity, and similar Fenton-like reactions occur with other transition metals such as copper [61]. These reactions, initially applied analytically, are now recognized as central to oxidative processes in biological and environmental systems [55,62]. Figure 2 illustrates the generation of ROS via the Fenton reaction in biological systems.

#### 1.2.2. Iron in Immune Function and Inflammation

Iron plays a dual role in immune regulation. Adequate iron supports lymphocyte proliferation, cytokine production, and macrophage function [63]. Iron-dependent enzymes are required for DNA synthesis during immune activation [63].

Conversely, pathogens require iron for growth and virulence. To limit microbial proliferation, the host reduces circulating iron levels during infection, a process mediated by inflammation-induced hepcidin upregulation [47,64]. While this mechanism restricts pathogen access to iron, chronic inflammation may lead to anemia of inflammation [64].

Recent studies also highlight iron’s role in ferroptosis, a regulated form of cell death. Ferroptosis is a distinct, non-apoptotic, iron-dependent process characterized by the accumulation of intracellular iron and the subsequent generation of ROS, which drives the peroxidation of polyunsaturated fatty acids in cellular membranes. This lipid peroxidation compromises membrane integrity and ultimately leads to cell death. Mechanistically, ferroptosis arises from the interplay among dysregulated iron metabolism, increased oxidative stress, and failure of antioxidant defense systems, particularly the glutathione–glutathione peroxidase 4 (GPX4) axis, resulting in unchecked lipid damage. Unlike apoptosis or necrosis, ferroptosis is caspase-independent and is primarily defined by iron-catalyzed oxidative membrane injury. Importantly, this pathway has been increasingly implicated in cancer biology, neurodegeneration, and inflammatory disorders, where it can exert both pathogenic and therapeutic roles [65,66].

#### 1.2.3. Iron and Neurocognitive Function

Iron is essential for normal brain development and neurotransmitter synthesis, serving as a cofactor for enzymes involved in dopamine, serotonin, and norepinephrine production [67]. Adequate iron levels during infancy and childhood are crucial for myelination and synaptic development [67].

Iron deficiency in early life has been associated with impaired cognitive performance and developmental delays [67]. Conversely, iron accumulation in specific brain regions has been linked to oxidative stress and neurodegenerative diseases, including Alzheimer’s and Parkinson’s disease [68].

#### 1.2.4. Iron in Thyroid and Endocrine Function

Iron contributes to thyroid hormone synthesis by serving as a cofactor in thyroid peroxidase (TPO), a heme-dependent enzyme required for iodination of thyroglobulin. Iron deficiency has been associated with impaired thyroid hormone production and may exacerbate hypothyroid states, particularly in iodine-deficient populations [69].

#### 1.2.5. Iron and Cardiovascular Health

Iron deficiency is prevalent among patients with heart failure and is associated with reduced exercise capacity and poorer clinical outcomes. Clinical trials have demonstrated that intravenous iron therapy improves symptoms and quality of life in patients with iron-deficient heart failure [70].

Conversely, iron overload conditions such as hereditary hemochromatosis promote oxidative injury in cardiac tissue, potentially leading to cardiomyopathy and arrhythmias [71]. ROS generated by the Fenton reaction causes oxidative damage to lipids, proteins, and mitochondrial DNA, leading to mitochondrial dysfunction, impaired calcium handling, and reduced contractile function in cardiomyocytes. Over time, this oxidative injury can result in cardiomyopathy, arrhythmias, and heart failure. These findings support a U-shaped relationship between iron status and cardiovascular risk [72].

#### 1.2.6. Iron in Reproductive Health

Iron requirements increase during menstruation and pregnancy due to blood loss and fetal demands [43,73]. Iron deficiency during pregnancy is associated with preterm birth and impaired neurodevelopment in offspring. In males, iron contributes to spermatogenesis and mitochondrial energy metabolism; however, excessive iron may induce oxidative stress in reproductive tissues [73].

Together, growing clinical and molecular evidence highlights that iron plays an essential role in human physiology, but its levels must be carefully regulated. Iron supports oxygen transport, mitochondrial respiration, immune competence, endocrine function, and neurocognitive integrity [43,47]. However, its redox activity necessitates tight regulatory control to prevent oxidative damage and pathological iron accumulation [44]. Advances in hepcidin biology, iron-immune interactions, and ferroptosis pathways continue to refine our understanding of iron’s role in health and disease, informing both nutritional strategies and therapeutic innovations [74].

#### 1.2.7. Iron in DNA Synthesis

Iron plays a significant role in DNA synthesis, primarily through its function as a cofactor for ribonucleotide reductase (RNR), the rate-limiting enzyme that converts ribonucleotides to deoxyribonucleotides, the building blocks required for DNA replication and repair. The catalytic activity of RNR depends on a stable diiron-tyrosyl radical center in its R2 subunit, which generates the radical necessary for nucleotide reduction. Iron is also indispensable for the activity of DNA primase and several DNA polymerases, as well as for the iron-sulfur (Fe-S) cluster-containing helicases and nucleases involved in DNA strand unwinding and processing [75]. Beyond enzymatic roles, iron participates in the regulation of the cell cycle at the G1/S checkpoint, where adequate intracellular iron availability is required for cells to commit to DNA replication. Iron deficiency consequently results in impaired RNR activity, depletion of the deoxyribonucleotide pool, stalled replication forks, and accumulation of DNA damage [76]. Conversely, excess iron promotes oxidative stress through Fenton chemistry, generating hydroxyl radicals that cause single- and double-strand DNA breaks, base modifications, and cross-links [77]. This dual dependence on iron sufficiency makes cellular iron homeostasis a critical determinant of genomic stability, and it underlies the rationale for iron chelation as a strategy to inhibit the proliferation of rapidly dividing cancer cells that have disproportionately elevated iron demands.

#### 1.2.8. Iron in Cell Proliferation

Iron is a fundamental driver of cell proliferation through several interconnected mechanisms. Most critically, the absolute requirement of ribonucleotide reductase (RNR) for iron directly gates deoxyribonucleotide synthesis and therefore DNA replication, creating an obligatory link between intracellular iron status and the ability to enter and complete S phase. Iron also sustains the mitochondrial electron transport chain by being present in cytochromes, Fe-S cluster complexes, and Complex IV subunits, generating the ATP required to fuel the energetically demanding processes of biosynthesis and cell division. At the regulatory level, rapidly dividing cells upregulate transferrin receptor 1 (TfR1) through iron regulatory proteins (IRP1/IRP2), increasing iron import in proportion to proliferative demand [78]. Cancer cells exploit this dependency by characteristically overexpressing TfR1, downregulating ferritin-mediated storage, and exhibiting heightened reliance on iron for survival, a state sometimes termed “iron addiction” [79]. This vulnerability has been therapeutically leveraged, as iron chelators such as deferoxamine can selectively arrest proliferation in iron-dependent tumor cells by depleting the labile iron pool required to sustain RNR activity and mitochondrial function [78,79].

#### 1.2.9. Iron in Cancer Biology

Iron plays a complex and critical role in cancer biology, largely because of its involvement in essential cellular processes, including DNA synthesis, oxygen transport, and energy metabolism. Cancer cells often exhibit dysregulated iron metabolism, increasing iron uptake while reducing its export to support rapid proliferation, a phenomenon widely described in the literature [80]. This excess intracellular iron can promote tumor growth by fueling enzymes needed for cell division and by generating reactive oxygen species (ROS), which can cause DNA damage and contribute to genetic instability. At the same time, iron can also trigger a form of cell death known as Ferroptosis, which has become a promising target in cancer therapy [81]. Researchers are actively exploring strategies to manipulate iron levels either by depriving cancer cells of iron or by enhancing iron-mediated toxicity to develop more effective treatments.

#### 1.2.10. Iron Toxicity and Overdose

Iron toxicity arises when iron accumulation exceeds the buffering capacity of transferrin and ferritin, allowing redox-active non-transferrin-bound iron (NTBI) to circulate freely and enter cells via unregulated uptake pathways. Once inside cells, free ferrous iron participates in the Fenton reaction, generating highly reactive hydroxyl radicals that induce lipid peroxidation, protein oxidation, and DNA strand breaks, collectively driving oxidative stress-mediated cellular injury [82]. In acute iron overdose, most commonly from accidental or intentional ingestion of pharmacological iron preparations, serum iron concentrations typically peak within 2 to 6 h, saturating transferrin and releasing NTBI into circulation. Free iron then concentrates in mitochondria, disrupting oxidative phosphorylation, impairing ATP production, and ultimately triggering cell death, with severe toxicity defined at elemental iron ingestion above 60 mg/kg [83]. The liver is the primary organ of iron accumulation and therefore sustains the greatest injury, but the heart, kidneys, pancreas, and endocrine organs are also vulnerable to progressive iron deposition. Chronic iron overload as seen in hereditary haemochromatosis, repeated transfusions in thalassemia and sickle cell disease, or excessive supplementation leads to cirrhosis, cardiomyopathy, diabetes mellitus, hypogonadism, and arthropathy through the same oxidative mechanisms, albeit at a slower tempo [82,83]. These systemic consequences underscore why precise regulation of iron homeostasis is essential and why iron excess, like iron deficiency, constitutes a clinically significant pathological state.

The iron physiology outlined in this section is directly relevant to understanding the biological fate and safety profile of iron nanoparticles administered for drug delivery. Following cellular internalization, IONPs are trafficked to lysosomes, where the acidic environment promotes their gradual degradation and the release of free Fe^2+^ and Fe^3+^ ions into the intracellular labile iron pool [84,85]. This released iron becomes subject to the same homeostatic mechanisms described above: it can be sequestered in ferritin, exported via ferroportin, or bound to transferrin for systemic redistribution, and its accumulation may trigger hepcidin upregulation as a compensatory response to prevent iron overload [86,87,88]. At clinically relevant doses, this additional iron burden is generally well-tolerated in healthy individuals due to the buffering capacity of these regulatory systems; however, in patients with pre-existing iron dysregulation, such as hereditary hemochromatosis, anemia of chronic disease, or compromised renal function, the cumulative iron load from IONP degradation may carry greater clinical significance and warrants careful monitoring [89,90].

Furthermore, the Fe^2+^ ions released during nanoparticle degradation can participate in Fenton chemistry, generating reactive oxygen species that, while therapeutically exploited in ferroptosis-based cancer strategies, may simultaneously contribute to oxidative stress in healthy surrounding tissues. This duality underscores why a thorough understanding of systemic iron regulation is not merely background knowledge but a prerequisite for the rational design, dosing, and safety evaluation of iron-based nanoparticle drug delivery systems.

### 1.3. Iron Nanoparticles

An emerging and highly promising class of nanomaterials, iron-based nanoparticles, particularly IONPs, have attracted substantial attention for their multidimensional applications in biomedicine [91,92]. Owing to their superparamagnetic properties, tunable size, high surface-to-volume ratio, and favorable biocompatibility, iron nanoparticles offer unique advantages in diagnostic and therapeutic platforms [93]. Unlike bulk iron materials, iron nanoparticles exhibit physicochemical characteristics that enable precise interaction with biological systems, including enhanced cellular uptake, magnetic responsiveness, and controllable biodistribution [29].

Several clinically approved iron nanoparticle products have demonstrated the translational potential of superparamagnetic IONPs in medicine. Ferumoxytol, marketed as Feraheme (U.S.) and Rienso (EU), is an ultrasmall superparamagnetic IONP approved for intravenous treatment of iron deficiency anemia and has seen extensive off-label use as an MRI contrast agent due to its favorable relaxivity and safety profile. In 2025, the U.S. Food and Drug Administration also approved a dedicated imaging formulation, Ferabright™ (ferumoxytol injection), as the first iron-based MRI contrast agent for brain tumor visualization. Other iron oxide-based MRI contrast agents, such as Resovist^®^ (ferucarbotran) and older products like Endorem^®^, Combidex^®^, and Lumirem^®,^ were clinically used in liver, lymph node, and gastrointestinal imaging, respectively, illustrating the broad clinical utility and translational progress of iron nanoparticles in diagnostic and therapeutic applications [94,95].

One of the most promising therapeutic strategies involving IONPs is magnetic hyperthermia therapy. In this approach, IONPs generate localized heat when exposed to an alternating magnetic field, resulting in selective destruction of cancer cells through apoptosis or ferroptosis while sparing surrounding healthy tissues [96,97,98]. Several recent studies have demonstrated that IONPs exhibit high magnetic heating efficiency and can be engineered to optimize heat generation, tumor targeting, and biocompatibility [99]. Furthermore, surface-modified or encapsulated IONPs show improved stability and cellular uptake, which are critical factors for effective therapeutic performance [100].

Iron nanoparticles generally demonstrate favorable bioavailability and biodegradability when appropriately surface-modified, as their degradation products can enter endogenous iron metabolic pathways, thereby reducing long-term toxicity concerns compared with non-biodegradable nanomaterials [101]. Furthermore, IONPs enable controlled and targeted drug release under external magnetic fields or in response to stimuli in the tumor microenvironment [102]. Their magnetic properties allow site-specific accumulation at pathological tissues, minimizing systemic exposure and enhancing therapeutic efficacy [103].

In addition to their delivery capabilities, iron nanoparticles participate in redox-related biological processes. Through Fenton and Haber–Weiss reactions, iron catalyzes the generation of ROS, which may be harnessed therapeutically to induce oxidative stress-mediated apoptosis in malignant cells [44]. This redox activity has been exploited in ferroptosis-based cancer therapy, where iron-catalyzed lipid peroxidation leads to the accumulation of reactive oxygen species and oxidative damage to cellular membranes, ultimately inducing selective tumor cell death [104,105,106]. Importantly, when carefully engineered with polymeric coatings or targeting ligands, iron nanoparticles can maintain acceptable safety profiles while maximizing therapeutic benefit [101].

Following cellular internalization, IONPs are trafficked through early endosomes to late endosomes and ultimately to lysosomes, where the acidic environment (pH 4.5–5.5) promotes nanoparticle degradation and the release of iron ions. Recent proteomic analysis of dextran-coated IONPs in human tumor cells has demonstrated that the protein fingerprint of exocytosed particles serves as a molecular marker of the intracellular trafficking route taken, revealing time-dependent lysosomal exocytosis as a predominant clearance mechanism [102]. This lysosomal processing is both a therapeutic opportunity, enabling pH-triggered drug release, and a potential liability, as sustained lysosomal iron accumulation may overwhelm ferritin buffering capacity and contribute to oxidative stress, as discussed in Section 1.2.1. For drug delivery applications, the design challenge is therefore to engineer surface coatings that extend circulation, preserve targeting functionality despite corona formation, and modulate intracellular iron release kinetics to maximize therapeutic effect while limiting off-target oxidative injury.

Due to these properties, iron nanoparticles serve dual roles as both drug carriers and active therapeutic agents. Their ability to enhance MRI contrast further establishes them as theranostic platforms integrating diagnosis and treatment [92]. This multifunctionality distinguishes iron nanoparticles from many conventional nanocarriers.

### 1.4. Iron Nanoparticles in Drug Delivery

**Figure 3:**
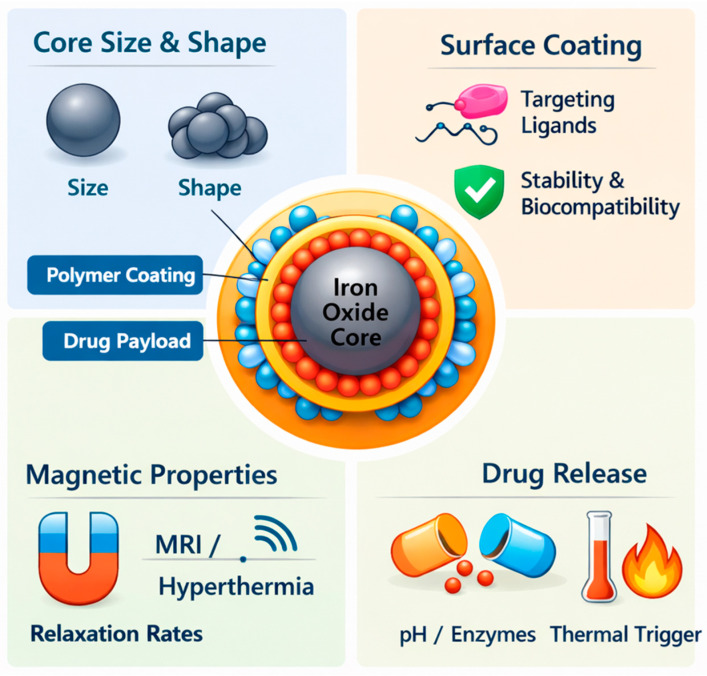
Structural architecture and multifunctional behavior of IONPs. The superparamagnetic Fe_3_O_4_/γ-Fe_2_O_3_ core enables magnetic responsiveness, while surface functionalization supports stability, targeting, and drug loading through multiple mechanisms, enabling stimuli-responsive therapeutic and diagnostic applications.

Iron Nanoparticles have emerged as highly promising nanomaterials in drug delivery and medical theranostics due to their unique physicochemical and magnetic properties [98,100]. Herein, we discuss the applications of IONPs, Zero-Valent Iron Nanoparticles (nZVI), and Iron-Based Core–Shell Nanoparticles in drug delivery. We also provide an overview of key properties of IONPs that make them appropriate for drug delivery. The structural features and multifunctional behavior of IONPs are summarized in Figure 3.

#### 1.4.1. IONPs

The primary objective of incorporating IONPs into drug delivery systems is to develop safer and more efficient cancer therapies. Magnetic nanoparticles can transport therapeutic agents to tumor tissues, allowing controlled drug release and improved therapeutic efficacy compared with conventional systemic chemotherapy [98,107]. This targeted delivery strategy has the potential to significantly reduce the adverse side effects commonly associated with cytotoxic medications while improving treatment outcomes for patients with difficult-to-treat cancers. Magnetite (Fe_3_O_4_) and Maghemite (γ-Fe_2_O_3_) are examples of IONPs.

Magnetite (Fe_3_O_4_) is a mixed-valence iron oxide nanoparticle composed of both Fe^2+^ and Fe^3+^ ions, which gives it strong superparamagnetic properties. Due to its high magnetic responsiveness, biocompatibility, and relatively low toxicity, magnetite nanoparticles are widely used in biomedical applications such as targeted drug delivery, MRI, and magnetic hyperthermia [101,108].

Jia et al. (2012) [109] developed a biodegradable, magnetically responsive drug delivery system in which Fe_3_O_4_ nanoparticles and Doxorubicin (Dox) were co-encapsulated within PLGA nanocarriers for intratumoral therapy. The nanoparticles had an average size of approximately 200–300 nm (≈280 nm) with a high encapsulation efficiency of ~90% and drug loading of ~85 µg/mg. They exhibited sustained and pH-sensitive release behavior, with about 65% of the drug released under acidic conditions (pH 5.0) compared to only ~25% at physiological pH (7.4) over 7 days. In vitro studies showed efficient cellular uptake and significantly enhanced apoptosis, with ~80% cell death observed for the nanoparticle formulation versus ~29% for free drug. In vivo, a single intratumoral dose (10 mg/kg) resulted in markedly improved antitumor efficacy, where tumor volume decreased to ~1.03 cm^3^ with magnetic targeting compared to ~1.91 cm^3^ for free drug and ~2.31 cm^3^ in controls after 14 days. Overall, the system demonstrated enhanced tumor retention, reduced metastasis, and improved therapeutic outcomes, especially when combined with an external magnetic field [109].

Another example is Maghemite (γ-Fe_2_O_3_), which is an oxidized form of magnetite containing only Fe^3+^ ions arranged in a defect spinel crystal structure. It exhibits excellent chemical stability, superparamagnetic behavior, and good biocompatibility, making it suitable for applications in drug delivery systems, biosensing, and magnetic seeds [91,110].

Wu et al. [111] presented a strategy for the synthesis of superparamagnetic γ-Fe_2_O_3_ nanoparticles encapsulated within metal–organic frameworks (MOFs) using an in situ pyrolysis method. Ferric triacetylacetonate was incorporated into MOFs such as Zeolitic imidazolate framework-8 (ZIF-8) and Materials of Institute Lavoisier-53, Aluminum version (MIL-53(Al)) and thermally decomposed to form γ-Fe_2_O_3_ nanoparticles uniformly distributed within the porous framework while preserving the crystallinity and high surface area of the MOFs. The resulting composites exhibited superparamagnetic behavior with moderate saturation magnetization, allowing magnetic manipulation without aggregation. To evaluate their biomedical potential, γ-Fe_2_O_3_@MIL-53(Al) was used as a carrier for ibuprofen delivery, demonstrating efficient drug loading and controlled release over approximately seven days in physiological conditions.

- Key Properties of IONPs for Drug Delivery

1. Superparamagnetic Properties

A key feature that makes IONPs particularly suitable for drug delivery applications is their superparamagnetic behavior. The magnetic responsiveness of Superparamagnetic iron oxide nanoparticles (SPIONs) enables magnetic targeting, in which drug-loaded nanoparticles can be guided to a specific disease site using an externally applied magnetic field. This strategy allows localized drug accumulation in target tissues, such as tumors, thereby enhancing therapeutic efficacy while minimizing systemic toxicity and side effects [7].

SPIONs, including magnetite (Fe_3_O_4_) and maghemite (γ-Fe_2_O_3_), exhibit magnetization only when exposed to an external magnetic field and lose it immediately upon removal of the field [112]. This behavior occurs because nanoparticles with sufficiently small sizes typically exist as single magnetic domains, preventing permanent magnetization.

One major advantage of superparamagnetism is the absence of remanent magnetization, which prevents irreversible aggregation of nanoparticles once the external magnetic field is removed. This property significantly improves colloidal stability in physiological environments and reduces the risk of particle clustering in biological systems [113]. Because of these advantages, SPIONs have become promising platforms for targeted drug delivery [114].

However, the translational effectiveness of magnetic targeting is not without limitations. A critical performance challenge is the attenuation of magnetic field strength with increasing tissue depth, which limits the effective magnetic guidance to superficial or surgically accessible tumors. For deep-seated malignancies, such as pancreatic, ovarian, or brain tumors, externally applied fields may be insufficient to overcome blood flow drag forces and achieve meaningful nanoparticle accumulation at the target site [115]. Addressing this limitation has driven the development of high-gradient magnet systems, implantable compared to IONPs, and the use of alternating magnetic fields in hyperthermia-based strategies [116,117], each representing engineering trade-offs between targeting precision, clinical feasibility, and patient safety. These constraints must be considered when evaluating the real-world drug delivery performance of SPIONs and inform the design criteria for next-generation magnetic targeting systems.

It is important to note that the majority of published magnetic targeting studies demonstrating high tumor accumulation have been conducted in subcutaneous xenograft models, where the tumor is near the skin surface and therefore accessible to clinically achievable field gradients. The translational gap between these idealized preclinical models and the clinical reality of deeply embedded pancreatic, ovarian, or brain tumors remains a fundamental and insufficiently acknowledged constraint in the IONP drug delivery literature. In vivo evidence suggests that, without magnetic field assistance, passive EPR-mediated tumor accumulation accounts for only a small fraction of the injected nanoparticle dose, on average across tumor models. These constraints reinforce the argument that active targeting strategies, receptor-mediated, or cell membrane-based, are not merely complementary to magnetic guidance but are, in many clinical contexts, the primary driver of specificity [118].

2. Biocompatibility

Another important advantage of IONPs for drug delivery is their excellent biocompatibility and biodegradability. Iron is a naturally occurring element in the human body and plays a crucial role in several physiological processes, including oxygen transport, enzymatic reactions, and cellular metabolism. After administration, IONPs can gradually degrade in biological environments, releasing iron ions that enter the body’s normal iron metabolic pathways. These ions can subsequently be incorporated into endogenous iron pools and utilized in physiological processes without causing significant toxicity when administered at appropriate doses [101,119].

Once degraded, the released iron can be stored in ferritin or incorporated into hemoglobin and other iron-containing proteins, allowing the body to efficiently recycle the material. This natural metabolic integration distinguishes IONPs from many other inorganic nanomaterials that may accumulate in tissues and cause long-term toxicity. Studies have shown that IONPs are typically processed by the reticuloendothelial system (RES), particularly in organs such as the liver and spleen, where macrophages metabolize the particles and regulate iron homeostasis [18,110]. Because of this intrinsic compatibility with biological systems, IONPs are considered promising and relatively safe platforms for drug delivery.

Nevertheless, biocompatibility is dose-dependent and context-dependent rather than an absolute property. As discussed in Section 1.2.1, Fe^2+^ ions released during IONP degradation can participate in Fenton chemistry, catalyzing the conversion of hydrogen peroxide to highly reactive hydroxyl radicals (OH•). At elevated or repeated doses, or in tissues with impaired antioxidant defenses, this iron-catalyzed ROS generation may contribute to oxidative damage to lipids, proteins, and nucleic acids [120,121]. This dose-dependent pro-oxidant risk is particularly relevant in the liver and spleen, the primary sites of RES-mediated IONP clearance, where chronic iron accumulation may promote hepatocellular oxidative stress. While this Fenton reactivity is therapeutically exploited in ferroptosis-based cancer strategies [122], it underscores the need for biocompatibility assessments to account for cumulative iron burden, dosing frequency, and the redox status of the target tissue. These considerations reinforce the importance of surface engineering strategies, such as antioxidant polymer coatings, designed not only to extend circulation time but also to modulate the rate of iron ion release and limit off-target oxidative injury.

3. Surface Functionalization

Surface functionalization is another key property that makes IONPs highly suitable for drug delivery applications. The surface of IONPs can be readily modified with a variety of organic polymers, biomolecules, and targeting ligands, thereby improving stability, biocompatibility, and biological performance. Common coating materials include polyethylene glycol (PEG), chitosan, dextran, peptides, and antibodies, which are used to tailor the physicochemical and biological characteristics of the nanoparticles for specific therapeutic applications [17,110]. These coatings not only protect the magnetic core from oxidation and aggregation but also provide functional groups that enable further conjugation of drugs or targeting molecules.

Among these coatings, PEGylation is widely used because PEG creates a hydrophilic steric barrier around the nanoparticle surface, thereby reducing protein adsorption and immune recognition. This “stealth” property minimizes uptake by the RES and significantly prolongs circulation time in the bloodstream [110,119]. Similarly, natural polymers such as dextran and chitosan have been extensively employed to stabilize IONPs due to their excellent biocompatibility and ability to provide reactive functional groups for drug conjugation. Dextran-coated IONPS have been widely investigated for biomedical applications, including imaging and drug delivery, while chitosan coatings can enhance muco-adhesion and improve drug-loading capacity [17,123].

In addition to polymer coatings, IONPs can also be functionalized with peptides or antibodies, enabling active targeting toward specific cells or tissues. These biomolecules recognize and bind to overexpressed receptors on diseased cells, such as cancer cells, allowing the nanoparticles to selectively accumulate at the target site. This strategy improves target specificity, enhances therapeutic efficiency, and reduces off-target toxicity. Consequently, surface-functionalized IONPs represent versatile platforms for targeted drug delivery, combining prolonged circulation, reduced RES clearance, and selective interaction with disease biomarkers [17,123].

While the coatings described above each offer distinct advantages, a direct comparison reveals important trade-offs in pharmacokinetics, colloidal stability, targeting capability, and clinical suitability that must be considered when designing iron nanoparticle systems for specific therapeutic contexts. Table 1 summarizes these comparative properties across the major surface-coating strategies used for IONPs in drug delivery.

An increasingly prominent strategy in IONP surface engineering is the coating of nanoparticles with natural cell membranes, including those derived from red blood cells (RBCs), cancer cells, macrophages, platelets, and T cells. This biomimetic approach confers several advantages over synthetic polymer coatings: cell membrane-coated IONPs inherit the surface protein repertoire of the donor cell, enabling immune evasion via CD47 “do not-eat-me” signaling, prolonged systemic circulation, and, in the case of homotypic cancer cell membranes, selective accumulation at tumor sites through homologous adhesion mechanisms [124]. RBC membrane-coated SPIONs have demonstrated significantly extended circulation half-lives compared to PEGylated counterparts, while macrophage membrane coatings exploit natural tumor-homing behavior to improve intratumoral accumulation. Cell membrane-coated IONPs loaded with ferroptosis-inducing agents have shown enhanced GPX4 inhibition and lipid peroxidation in hepatocellular carcinoma models, demonstrating the convergence of biomimetic targeting with iron-catalyzed therapeutic mechanisms [125]. Despite these advantages, biomimetic membrane coating presents practical challenges, including complex, low-yield fabrication processes, the risk of immunogenic transfer of membrane proteins, and batch-to-batch variability. Nevertheless, cell membrane-coated IONPs represent a compelling frontier in iron nanoparticle drug delivery that bridges nanotechnology and cell biology, and their continued development is expected to play a significant role in advancing clinical translation.

**Table 1:** Comparison of common surface coating strategies for IONPs in drug delivery.

Coating Type	Key Properties	Advantages	Limitations	References
PEG	Hydrophilic, flexible synthetic polymer	Prolonged circulation; reduced protein adsorption and RES uptake; “stealth” effect	Reduced cellular uptake at the target site	[110,119,126]
Dextran	Natural polysaccharide; reactive hydroxyl groups	High biocompatibility; reactive surface groups for further functionalization	Relatively rapid renal clearance; limited active targeting without additional ligands	[17,123]
Chitosan	Cationic natural polysaccharide; pH-responsive	Enhanced mucoadhesion; high drug loading capacity; pH-responsive release in acidic tumor microenvironments	Tendency to aggregate at physiological pH	[17,123]
Silica (SiO_2_)	Inorganic mesoporous shell; chemically stable	High chemical stability; tunable pore size for controlled drug loading; easy surface functionalization	Potential long-term accumulation in tissues	[127,128]
Peptides/Antibodies	Biomolecular targeting ligands	High receptor specificity; active targeting via receptor-mediated endocytosis; selective tumor accumulation	High production cost; potential immunogenicity; complex conjugation chemistry	[17,110]
Cell Membrane	Biomimetic natural membrane (RBC, cancer cell, macrophage, platelet)	Immune evasion via CD47 “do not-eat-me” signaling; prolonged systemic circulation; homotypic tumor targeting	Complex fabrication process; batch-to-batch variability; risk of immunogenic protein transfer	[124,125]

#### 1.4.2. Zero-Valent Iron Nanoparticles (nZVI)

Another example of Iron Nanoparticles is nZVI. Fe(0) nanoparticles exhibit a favorable combination of biocompatibility and chemical versatility, making them promising materials for a wide range of advanced applications, including anticancer activity, targeted drug delivery, contrast imaging, and environmental remediation [11,129].

Wu et al. [130] documented that the integration of nZVI nanoparticles with ferroptosis-inducing agents creates a promising therapeutic strategy for cancer treatment. In this approach, ZVI-based nanoparticles enhance the delivery of ferroptosis inducers to tumor tissues through the enhanced permeability and retention (EPR) effect, allowing them to preferentially accumulate in tumor lesions due to the leaky vasculature and poor lymphatic drainage in tumor microenvironments. Moreover, ZVI nanoparticles can contribute to synergistic anticancer activity by promoting iron-dependent lipid peroxidation and ROS generation, key mechanisms involved in ferroptosis. As a result, the combination of ZVI nanoparticles and ferroptosis-inducing compounds improves therapeutic efficacy by increasing drug accumulation at tumor sites while simultaneously amplifying ferroptotic cell death pathways in cancer cells. It should be noted that, compared to IONPs, nZVIs have been explored much less extensively in the field of drug delivery and currently exhibit relatively limited biomedical applications [131,132,133,134,135].

Sharma et al. [136] reported the development of zero-valent iron nanoparticles (ZVINPs) incorporated into high-density gastroretentive pellets to enhance oral iron bioavailability. The synthesized nanoparticles exhibited a particle size in the range of ~100–300 nm and a zeta potential of approximately 24 mV, indicating good stability and dispersion. The optimized pellet formulation demonstrated favorable micromeritic properties, including a bulk density of ~2.09 g/cm^3^ and negligible friability, enabling prolonged gastric retention of up to 10 h. In vitro release studies showed sustained iron release for up to 24 h, with ZVINPs reaching maximum release at ~19 h compared to ~21 h for ferrous sulfate. Pharmacokinetic analysis in Wistar rats revealed a significantly higher C max for ZVINPs (45.1 ± 5.53 mg/mL) compared to marketed ferrous sulfate (31.23 ± 2.34 mg/mL), along with an extended mean residence time for gastroretentive pellets (52.26 ± 9.82 h). Importantly, the formulation achieved more than a twofold increase in oral bioavailability relative to conventional iron therapy. While acute toxicity studies showed no significant hepatic damage, chronic administration led to elevated iron accumulation in organs such as the liver and pancreas and increased pancreatic enzyme levels, indicating potential dose-dependent toxicity that warrants further investigation.

#### 1.4.3. Iron-Based Core–Shell Nanoparticles

Another group of iron nanoparticles is iron-based core–shell nanoparticles. These nanoparticles have attracted considerable attention as nanocarriers for drug delivery because their architecture combines a magnetic iron or iron oxide core with a functional outer shell that improves stability and biocompatibility. The outer shell, commonly composed of polymers, silica, lipids, or other biocompatible materials, facilitates drug loading, surface modification with targeting ligands, and controlled drug release, enhancing the therapeutic performance of the nanocarrier. Moreover, the magnetic core enables magnetically guided targeting and accumulation at disease sites, thereby improving drug delivery efficiency while reducing systemic toxicity [13,29]. Specific examples are provided below:

##### Gold Shell (Fe_3_O_4_@Au)

Earlier, Salikhov et al. also extensively elaborated on the advances in the synthesis of Fe_3_O_4_@Au core/shell nanoparticles [137]. Hakimian et al. [138] reported the synthesis of multifunctional Fe_3_O_4_/Au/porous Au nanohybrids possessing both magnetic properties and high porosity for drug delivery applications. In their study, IONPs were first synthesized and then coated with a gold layer to enhance their chemical stability. To further increase the drug-loading capacity, a porous gold shell was formed on the Fe_3_O_4_/Au surface by creating an Ag–Au nanohybrid layer followed by selective dissolution of silver using dilute HNO_3_ (0.01 M). Dynamic light scattering analysis revealed that the synthesized nanohybrids had an average particle size of 68.0 ± 7.7 nm and a zeta potential of −28.1 ± 0.2 mV, indicating good colloidal stability. The anticancer drug DOX was successfully loaded onto the nanocarriers, and the DOX-loaded Fe_3_O_4_/Au/porous Au nanoparticles exhibited enhanced therapeutic efficacy against MCF-7 cancer cells compared with free DOX, showing approximately a 1.5-fold improvement in antitumor activity.

##### Silica Coating (Fe_3_O_4_@SiO_2_)

Fang et al. reported [139] the development of multifunctional DOX/MNPs-folic acid nanocomposites designed for simultaneous tumor imaging and targeted drug delivery. These core–shell nanoparticles comprised a magnetic iron oxide core with tunable magnetism via Zn^2+^ doping, enabling them to serve as efficient T_2_-weighted MRI contrast agents. The nanoparticles were then coated with a porous silica shell with large pores (~5.4 nm), providing a high surface area for efficient loading of the anticancer drug DOX. Drug release from the nanocarrier was triggered under acidic conditions (pH 4–6), mimicking the tumor microenvironment. Additionally, surface functionalization with folic acid enabled receptor-mediated targeting of tumor cells. The in vitro studies demonstrated enhanced cellular uptake in HeLa cells via folate receptor-mediated endocytosis and significantly greater cytotoxicity than free DOX or non-targeted nanoparticles.

##### Polymer Coatings

Polymer-coated Fe_3_O_4_ nanoparticles have been widely explored for drug delivery applications due to their improved stability, biocompatibility, and ability to carry therapeutic molecules. The polymer layer acts as a protective coating around the magnetic Fe_3_O_4_ core, preventing aggregation, reducing toxicity, and enhancing circulation time in the bloodstream. Commonly used polymers include PEG, poly(lactic-co-glycolic acid (PLGA), chitosan, and polyvinyl alcohol (PVA), which provide functional groups that facilitate drug loading and surface modification with targeting ligands. In addition, polymer coatings enable controlled and sustained drug release while maintaining the magnetic properties of the Fe_3_O_4_ core for magnetic targeting and imaging [140,141].

Jain et al. [142] developed water-dispersible oleic acid (OA)-Pluronic-coated IONPs capable of efficiently loading hydrophobic anticancer drugs. The drug was partitioned into the oleic acid shell surrounding the iron oxide core, while the Pluronic coating provided stability and dispersibility in aqueous environments without affecting the magnetic properties of the nanoparticles. These nanoparticles exhibited sustained drug release for up to two weeks and demonstrated dose-dependent antiproliferative effects in breast and prostate cancer cell lines, suggesting their potential for magnetic targeting and drug delivery.

In addition, Yallapu et al. [143] reported the development of IONPs coated with oleic acid and OA-PEG to produce water-dispersible carriers capable of loading hydrophobic DOX for sustained drug delivery. The nanoparticles, with a hydrodynamic diameter of approximately 184 nm and an 8 nm iron oxide core, enhanced T_2_ MRI contrast and exhibited prolonged circulation in vivo. Furthermore, DOX-loaded nanoparticles demonstrated sustained drug release and dose-dependent antiproliferative effects in breast cancer cells, with improved targeting when transferring antibodies were conjugated to the nanoparticle surface.

Recently, Ozkahraman et al. [144] developed pH-responsive chitosan-coated iron oxide nanoflower (IONF) nanogels for controlled delivery of DOX in cancer therapy. The chitosan coating provided biocompatibility, stability, and pH-responsive behavior, enabling enhanced drug release under acidic tumor conditions compared with physiological pH. The nanocarriers exhibited high drug loading capacity (~67%) and loading efficiency (~84%), while maintaining the superparamagnetic properties of the iron oxide core. In vitro studies demonstrated effective cellular uptake and significant cytotoxicity against cancer cells upon DOX loading, whereas the unloaded nanocarriers showed minimal toxicity, suggesting the potential of chitosan-functionalized magnetic nanoparticles as promising platforms for targeted and controlled anticancer drug delivery.

## 2. Drug Loading Strategies

**Figure 4:**
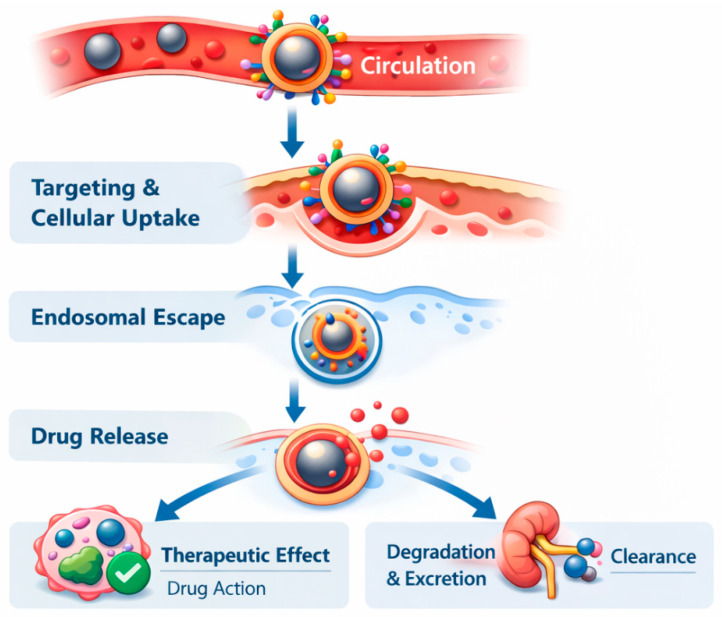
Mechanistic pathway of IONP-mediated drug delivery. Following systemic administration, nanoparticles undergo circulation, tumor targeting, cellular internalization, and stimuli-responsive drug release, leading to multimodal therapeutic effects and eventual biodegradation through endogenous iron metabolic pathways.

Drug loading in nanocarrier systems is primarily achieved through non-covalent and covalent strategies, each balancing loading efficiency, stability, and release control. Non-covalent approaches rely on physical encapsulation and weak interactions, such as electrostatic and hydrophobic forces, enabling high drug loading without chemical modification, though with less control over release. In contrast, covalent conjugation forms stable chemical linkages that improve circulation stability and allow stimuli-responsive, targeted drug release, albeit with typically lower loading efficiency and greater design complexity. Together, these approaches underpin the rational development of nanocarrier-based drug delivery systems. The major delivery pathways and intracellular mechanisms of IONP-mediated drug delivery are illustrated in Figure 4.

### 2.1. Non-Covalent Conjugation

Drug incorporation into nanocarriers can also occur through noncovalent interactions. In these systems, the therapeutic agent is associated with the carrier through physical encapsulation or weak intermolecular forces rather than covalent chemical bonds. The physicochemical characteristics of the nanocarrier, particularly particle size and morphology, play an important role in determining the extent of drug incorporation and stabilization within the carrier matrix. Through encapsulation, the drug is loaded into a suitable nanocarrier system, enabling its incorporation without the need for chemical modification of the therapeutic molecule [36,145].

One of the major methods to load the drug onto MNPs is using electrostatic interactions between the nanocarriers and the cargo drug. In addition, the presence of functional groups in the nanocarrier structure that generate hydrophobic forces will strengthen the binding affinity between the drug and the nanocarrier. We have extensively discussed these in our previous reports [32,33,34,35,36,37]. Similar approaches have been used for iron nanoparticles. Table 2 illustrates two specific examples.

**Figure 5:**
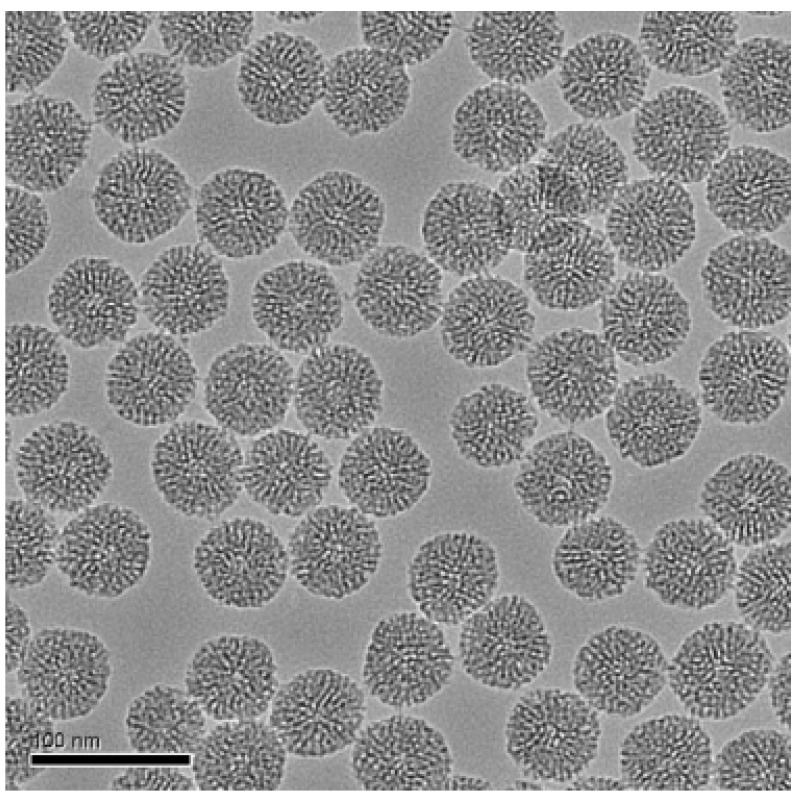
Enlarged transmission electron microscopy (TEM) images of H-mSiO_2_. Reprinted with permission from [146]. Copyright 2008 from John Wiley and Sons. The Figure was reprinted with permission from [146]—[Kim, J.; Kim, H.S.; Lee, N.; Kim, T.; Kim, H.; Yu, T.; Song, I.C.; Moon, W.K.; Hyeon, T.], [Multifunctional uniform nanoparticles composed of a magnetite nanocrystal core and a mesoporous silica shell for magnetic resonance and fluorescence imaging and for drug delivery]; published by [John Wiley and Sons], [2008].

Kim et al. [146] reported the synthesis of Fe_3_O_4_@mSiO_2_(R) nanoparticles followed by surface functionalization and drug loading for anticancer delivery. The nanoparticles were initially prepared by coating 15 nm Fe_3_O_4_ nanocrystals with a mesoporous silica shell using a cetyltrimethylammonium bromide (CTAB) templated sol–gel process involving tetraethylorthosilicate (TEOS) and rhodamine B isothiocyanate–APTES conjugates. The resulting Fe_3_O_4_@mSiO_2_(R) nanoparticles were subsequently functionalized with PEG through a covalent reaction between amine groups on the nanoparticle surface and the succinimidyl groups of methoxy-PEG-succinimidyl glutarate, forming stable Fe_3_O_4_@mSiO_2_(R)-PEG nanostructures that improved colloidal stability and biocompatibility. For drug incorporation, the PEG-functionalized nanoparticles were mixed with a DOX solution in phosphate-buffered saline and stirred overnight, allowing DOX molecules to be adsorbed into the mesoporous silica framework. After loading, the nanoparticles were collected by centrifugation, and the amount of unadsorbed DOX remaining in the supernatant was quantified using UV–visible spectroscopy to determine the loading efficiency. The resulting DOX-loaded Fe_3_O_4_@mSiO_2_(R)-PEG nanoparticles were then redispersed in water for further biological applications. Figure 5 shows enlarged TEM images of H-mSiO_2_.

Similar approaches were reported by other researchers as well [147,148,149,150]. Another popular method is encapsulation of the drug onto the IONPs. Cytarabine has been successfully incorporated into iron oxide–based nanocarriers via encapsulation. In one study by Singh et al. [151], IONPs were coated with casein using an emulsion crosslinking method combined with in situ precipitation to prevent surface oxidation and nanoparticle agglomeration. The resulting casein-coated IONPs (CCIONPs) possessed a core–shell morphology with particle sizes ranging from approximately 95 to 150 nm, as confirmed by transmission electron microscopy. Cytarabine was subsequently encapsulated within these biopolymer-coated nanoparticles, forming Cyt-loaded CCIONPs. The encapsulation system demonstrated significant anticancer activity in vitro, particularly against HepG2 liver cancer cells, where the nanoparticles inhibited colony formation, suppressed cell migration and invasion, and induced apoptosis.

**Table 2:** Comparison of non-covalent drug loading strategies for IONPs.

Nanoparticle System	Drug Loaded	Loading Method	Particle Size (nm)	Reference
Fe_3_O_4_@mSiO_2_(R)-PEG	Doxorubicin (DOX)	Physical adsorption into mesoporous silica pores	~15 (core)	[146]
Casein-coated IONPs (CCIONPs)	Cytarabine	Encapsulation via emulsion crosslinking + in situ precipitation	95–150	[151]

### 2.2. Covalent Conjugation

Covalent conjugation between a drug and its carrier provides several advantages in drug delivery. It improves the drug’s stability during circulation and prevents premature release. This approach also enables controlled and site-specific drug release under specific conditions, such as pH or enzymatic activity. As a result, it can enhance targeting efficiency and reduce systemic toxicity [113].

IONPs with drugs covalently attached to their surface often exhibit lower drug-loading efficiency and may face difficulties in achieving efficient drug release compared with systems based on noncovalent interactions. In addition, the chemical reactions used for covalent coupling can leave residual catalysts or reagents on the nanoparticle surface, which may contribute to in vivo toxicity. Another important consideration is that the drug’s molecular structure and orientation must remain unchanged during the conjugation process, as structural alterations may reduce or modify its biological activity [152]. Table 3 summarizes several covalent drug-conjugation strategies for IONPs.

**Figure 6:**
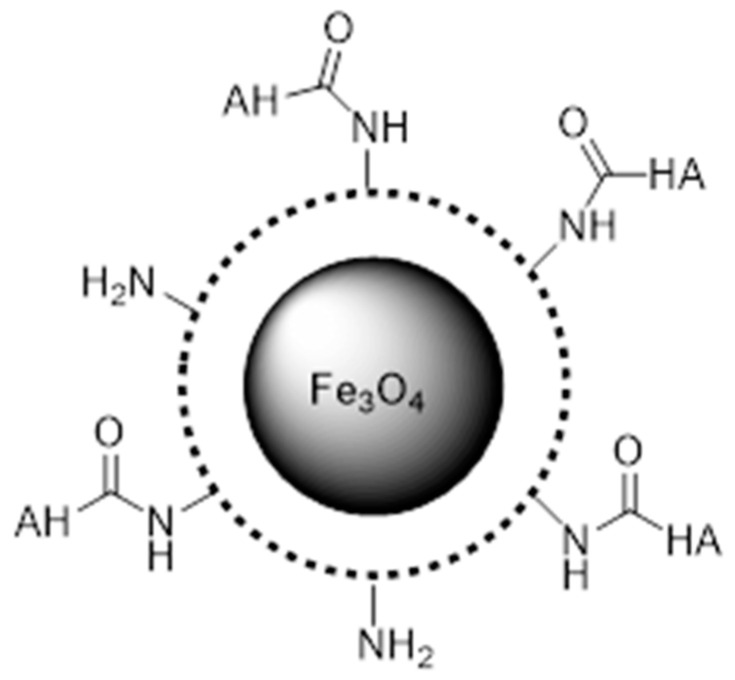
Hyaluronan (HA)-coated superparamagnetic iron oxide nanoparticles (HA-SPION). The Figure was reproduced with permission from [153]—[El-Dakdouki, M.H.; Zhu, D.C.; El-Boubbou, K.; Kamat, M.; Chen, J.; Li, W.; Huang, X.], [Development of Multifunctional Hyaluronan-Coated Nanoparticles for Imaging and Drug Delivery to Cancer Cells], published by [American Chemical Society], [2012].

For instance, El-Dakdouki et al. reported a covalent conjugation between hyaluronan (HA)-coated superparamagnetic iron oxide nanoparticles (HA-SPION) and DOX [153]. These nanoparticles exploit the interaction between hyaluronan (HA) and CD44, a receptor overexpressed on many tumor cells, to achieve selective uptake, with more than a tenfold enhancement in SKOV-3 ovarian cancer cells compared to uncoated SPIONs. HA-SPIONs exhibit high T2* magnetic relaxivity (r2* = 431 mM^−1^·s^−1^), enabling effective MRI of cancer cells. Doxorubicin (DOX) was conjugated to HA-SPIONs via an acid-sensitive hydrazone linker, which remains stable at physiological pH but releases DOX under acidic lysosomal conditions. This design significantly enhanced cytotoxicity: the IC_50_ of DOX-HA-SPION was 0.079 μg/mL in SK-OV-3 cells and 17.5 μg/mL in multidrug-resistant NCI/ADR-RES cells, representing a 4-fold improvement over free DOX in the latter. Confocal imaging revealed that DOX delivered by HA-SPIONs initially accumulates in the cytosol and mitochondria before translocating to the nucleus, suggesting a mechanism that may overcome drug resistance. Overall, HA-SPIONs provide a promising theranostic platform, combining targeted drug delivery, MRI-based imaging, and enhanced potency against resistant cancer cells. Figure 6 shows a schematic image of HA-SPION.

In another example, Zhu et al. [154] developed a multifunctional superparamagnetic iron oxide (SPIO) nanocomposite by covalently conjugating the anticancer drug DOX and PEG to SPIO nanoparticles through pH-sensitive acylhydrazone linkages. The resulting DOX-SPIO nanocomposites demonstrated good aqueous stability while preserving strong magnetic properties, enabling magnetic targeting under an external magnetic field. Importantly, the acylhydrazone bonds were cleaved under acidic conditions similar to those in tumor microenvironments, triggering rapid drug release. This pH-responsive system also exhibited enhanced cellular uptake and improved antitumor activity compared with free DOX.

**Figure 7:**
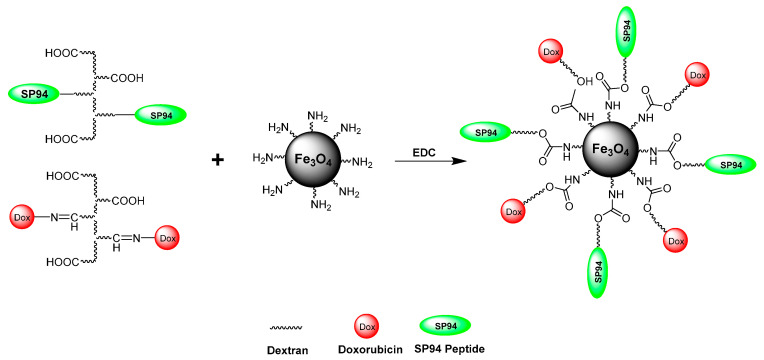
Schematic representation showing the synthesis of Fe_3_O_4_-DOX/SP94 multifunctional magnetic nanoparticles [155]. The Figure was reproduced with permission from [155]—[Wang, Y.; Jia, H.-Z.; Han, K.; Zhuo, R.-X.; Zhang, X.-Z.], [Theranostic magnetic nanoparticles for efficient capture and in situ chemotherapy of circulating tumor cells]; published by [The Royal Society of Chemistry], [2013].

Furthermore, Wang et al. [155] designed the Fe_3_O_4_-DOX/SP94 nanostructures through a multistep functionalization strategy involving dextran derivatives and aminated magnetic nanoparticles. Initially, Fe_3_O_4_ nanoparticles were prepared and modified with aminopropyltriethoxysilane (APTES) to introduce surface amine groups, producing Fe_3_O_4_-NH_2_ nanoparticles. Separately, carboxymethyl dextran (CMD) was synthesized and further modified to obtain dextran derivatives containing either the targeting peptide SP94 or the anticancer drug DOX. The peptide SP94 was grafted onto dextran through a click reaction between alkyne-modified dextran and azide-functionalized SP94, while DOX was conjugated to dextran via pH-sensitive hydrazone bonds. Finally, the multifunctional nanostructures were fabricated by coupling dex-SP94 and dex-DOX to Fe_3_O_4_-NH_2_ nanoparticles via 1-ethyl-3-(3-dimethylaminopropyl)carbodiimide (EDC)-mediated reactions between the amine groups on Fe_3_O_4_ and the carboxyl groups of the dextran derivatives, yielding Fe_3_O_4_-DOX/SP94 nanoparticles with targeting and drug delivery capabilities. Figure 7 represents a schematic representation showing the synthesis of Fe_3_O_4_-DOX/SP94 multifunctional magnetic nanoparticles [155].

## 3. Challenges and Limitations

Despite their promising properties, IONPs still face several challenges that limit their widespread clinical translation. One major issue is the scalability and reproducibility of synthesis methods. Common preparation techniques such as co-precipitation, thermal decomposition, and micro-emulsion synthesis can lead to batch-to-batch variability, affecting important characteristics, including particle size, magnetic behavior, and surface functionality, which are crucial for consistent drug loading and targeted delivery. Variations in these properties can influence the biodistribution, therapeutic efficacy, and overall safety of nanoparticles in biomedical applications [156].

Another challenge involves the long-term stability and biological performance of IONPs. During storage or in physiological environments, nanoparticles may aggregate or undergo surface oxidation, thereby reducing their effectiveness and altering their pharmacokinetics. Although surface functionalization and polymer coatings are often applied to improve stability and targeting capability, these modifications introduce additional complexity to the manufacturing process and require strict quality control to ensure ligand integrity and reproducibility [157].

Furthermore, comprehensive biocompatibility, biodistribution, and long-term toxicity studies are still needed to fully understand the biological fate of these materials. Regulatory pathways for multifunctional nanocarriers are also still evolving, creating uncertainty regarding clinical approval. Consequently, addressing these challenges will require standardized synthesis protocols, advanced characterization techniques, and collaborative efforts between materials scientists, pharmacologists, and regulatory agencies to enable the safe and effective use of iron nanoparticles in drug delivery [158]. A recent comprehensive review of the SPION clinical landscape confirms that while nanoparticle degradation, tissue-specific retention, and local delivery have enabled specific approved applications, these same properties simultaneously constrain SPIONs from serving as universal theranostic agents, underscoring the need for application-specific design strategies rather than one-size-fits-all platforms [26].

## 4. Future Directions

**Figure 8:**
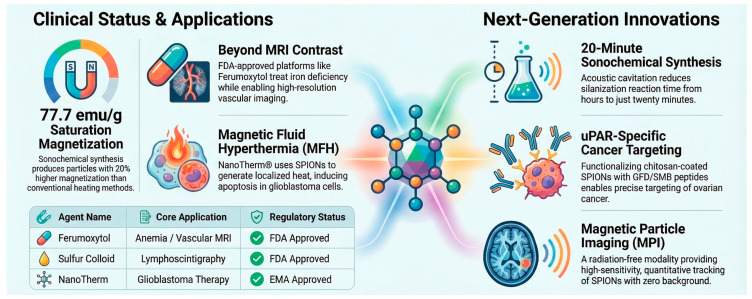
SPIONs: The Multi-Tool of Precision Medicine. SPIONs are biocompatible materials with unique magnetic properties. Their use ranges from MRI contrast to targeted cancer therapy, hyperthermia, and radiation-free molecular imaging, achieved via sonochemical synthesis and peptide functionalization. uPAR (urokinase-type plasminogen activator receptor).

The future of iron nanoparticles in drug delivery lies in their biocompatibility and their ability to serve as multifunctional nanoplatforms for imaging, targeted therapy, gene delivery, and combination treatments. By enabling early tumor detection, improving penetration through biological barriers, supporting precision medicine approaches, and delivering multiple drugs simultaneously, iron nanoparticles are expected to play a critical role in the next generation of nanomedicine-based cancer therapies. The following sections discuss their future potential according to the four categories shown in Figure 8.

### 4.1. Targeting Early Microscopic Tumors

Iron nanoparticles have strong potential to improve the early detection and treatment of microscopic tumors. SPIONs possess excellent magnetic properties, making them effective MRI contrast agents that allow clinicians to visualize small tumor lesions that may not be detectable with conventional imaging methods [159,160]. In future applications, these nanoparticles can be engineered to carry tumor-specific ligands, such as antibodies, peptides, or small molecules, that bind to biomarkers expressed on cancer cells. This selective targeting can promote the accumulation of iron nanoparticles in early-stage tumors while minimizing distribution in healthy tissues. In addition to enabling earlier diagnosis, these nanostructures can serve as theranostic platforms, providing imaging capabilities and delivering anticancer drugs directly to microscopic tumor sites. Such multifunctional systems may greatly enhance the effectiveness of early cancer management. Importantly, recent studies have demonstrated that combining iron nanoparticle-mediated ferroptosis with photothermal therapy and immune checkpoint blockade, for example, through cRGD/anisamide dual-targeted Fe_3_O_4_ nanoplatforms co-administered with anti-PD-L1, can achieve tumor inhibition rates exceeding 90% in colorectal cancer models by simultaneously inducing immunogenic cell death and activating systemic anti-tumor immunity, underscoring the convergence of early targeting, multimodal therapy, and immunotherapy as a defining direction for next-generation iron nanoparticle systems [161].

### 4.2. Overcoming Physical Barriers to Drug Delivery

Tumor tissues often contain dense extracellular matrices and abnormal vasculatures, which significantly limit the penetration of therapeutic agents. Iron nanoparticles offer several strategies to address these physical barriers. Because they respond to magnetic fields, externally applied magnetic forces can potentially guide magnetic nanoparticles toward tumor tissues, increasing drug accumulation at the desired site [162]. Moreover, iron nanoparticles can produce localized heating when exposed to alternating magnetic fields, a process known as magnetic hyperthermia. This localized heat can temporarily disrupt tumor structures and improve drug diffusion into deeper regions of the tumor [61]. Future designs may also incorporate stimuli-responsive coatings that trigger drug release in response to environmental conditions within the tumor microenvironment, such as acidic pH, enzymes, or temperature variations. These approaches may significantly improve the efficiency of drug delivery to otherwise difficult-to-reach tumor regions.

The challenge of deep-seated anatomical barriers, most critically the blood–brain barrier (BBB) and blood-tumor barrier (BTB) in glioblastoma, has prompted the development of combinatorial nanoparticle strategies that integrate magnetic field-guided delivery with focused ultrasound-mediated BBB disruption, cell-based carrier systems, and intranasal administration routes; together, these approaches offer synergistic improvements in drug penetration that no single modality can achieve alone, and their further development represents one of the most clinically significant frontiers in iron nanoparticle targeting [163].

### 4.3. Drug Delivery for Precision Medicine

Iron nanoparticles are also expected to play an important role in precision medicine, where therapies are tailored according to the genetic and molecular characteristics of individual patients. In this context, magnetic nanoparticles can function as carriers for gene-modulating molecules such as small interfering RNA (siRNA) or microRNA, enabling the regulation or silencing of genes associated with disease progression [164]. Additionally, these nanoparticles may facilitate the delivery of gene-editing systems such as CRISPR/Cas9, allowing targeted modification of specific genetic mutations. The magnetic properties of iron nanoparticles also allow them to be tracked using imaging techniques, enabling real-time monitoring of treatment distribution and effectiveness. Furthermore, iron nanoparticle platforms have the potential to support immunotherapy strategies, including the delivery of immune-stimulating agents designed to activate the body’s immune response against cancer cells. Such technologies may provide new approaches for addressing previously difficult or “undruggable” biological targets [165,166].

The full realization of precision nanomedicine with iron nanoparticles requires integration with the molecular and genomic characterization of individual tumors. In biomarker-driven targeting, surface ligands, peptides, antibodies, or aptamers are selected based on the receptor expression profile of a patient’s specific tumor subtype rather than generic cancer cell markers. For example, patients with HER2-overexpressing breast cancers, CD44-high sarcomas, or folate receptor-positive ovarian cancers represent molecularly defined subpopulations in whom receptor-targeted IONPs are expected to achieve substantially superior accumulation compared to unselected patient cohorts. Integration of tumor transcriptomic data, increasingly available through liquid biopsy and single-cell RNA sequencing, offers a roadmap for real-time adaptation of targeting ligand selection to the evolving receptor landscape of relapsed or treatment-resistant tumors. Furthermore, the convergence of IONP-mediated drug delivery with immunotherapy represents a clinically powerful direction: IONPs can simultaneously deliver checkpoint inhibitors, tumor-associated antigens, or immune adjuvants while inducing immunogenic cell death through Fenton-mediated ferroptosis, thereby converting immunologically ‘cold’ tumors into inflamed, immune-responsive lesions. Nano-drug delivery systems integrating these immunostimulatory mechanisms have demonstrated synergistic enhancement of antitumor immunity and durable immune memory responses in preclinical models, supporting their continued development as combination immuno-nanomedicine platforms [27].

### 4.4. Multidrug Delivery

Iron nanoparticles are particularly well suited for multidrug delivery systems, which aim to improve therapeutic outcomes by simultaneously targeting multiple disease pathways. Their large surface area and tunable surface chemistry enable them to simultaneously carry multiple therapeutic agents, including chemotherapeutic drugs, nucleic acids, and targeting ligands [7,93]. These agents can be incorporated using different loading strategies that enable controlled or sequential drug release, ensuring that each therapeutic compound is delivered at the most effective stage of treatment. By targeting multiple cellular pathways involved in tumor growth and progression, such combination therapies may help reduce the development of drug resistance [167].

In addition, iron nanoparticles can integrate multiple therapeutic modalities, such as chemotherapy combined with magnetic hyperthermia or diagnostic imaging, creating multifunctional theranostic platforms [168]. These capabilities position iron nanoparticles as promising tools for developing more effective and personalized cancer treatment strategies. Biocompatible polymeric nanoplatforms that integrate ferroptosis with chemotherapy, photodynamic therapy, photothermal therapy, and immunotherapy have demonstrated synergistic antitumor effects and durable immune memory responses against metastatic and drug-resistant cancers, representing a clinically promising extension of multidrug iron nanoparticle delivery strategies [169].

### 4.5. AI in Iron Nanoparticle Design

AI can be used to design and optimize iron nanoparticle-based drug delivery systems [120,121]. Traditional nanoparticle development often relies on extensive trial-and-error experimentation; however, AI and machine learning (ML) algorithms can analyze large experimental datasets to identify relationships between nanoparticle characteristics such as size, surface charge, coating materials, and magnetic properties and their biological performance, including drug loading capacity, stability, and cellular uptake. These computational tools enable researchers to predict optimal nanoparticle formulations and significantly accelerate the development process [164,170].

Recent studies have demonstrated that machine learning models can predict nanoparticle tumor delivery efficiency with high accuracy using physicochemical descriptors, including zeta potential and core material [171], map SPION toxicity landscapes based on surface chemistry parameters [172], and predict drug release properties across dozens of core-coating combinations [173], collectively establishing AI-driven design as a practical tool for accelerating IONP development [174].

More specifically, several ML model architectures have demonstrated practical utility for IONP design. Deep neural networks integrated with physiologically based pharmacokinetic (PBPK) modeling have achieved adjusted R^2^ values of 0.92 in predicting nanoparticle tumor delivery efficiency from physicochemical parameters, identifying zeta potential and core material as the dominant determinants of accumulation across tumor types [171]. Classification and regression models trained on SPION surface-chemistry descriptors, including coating type, charge, and functional group density, have successfully predicted toxicological outcomes and guided the formulation of safer SPION preparations with reduced cytotoxicity [172].

Multi-output ML models covering 54 different nanoparticle core classes and 25 coating types have demonstrated high sensitivity and specificity (>0.9) in predicting drug release profiles, cytotoxic concentrations, and cell-line-specific inhibition rates, substantially reducing trial-and-error experimental burden [174]. Generative AI and reinforcement learning approaches are also beginning to be applied to inverse nanoparticle design, working backward from a desired biodistribution or therapeutic outcome to propose novel core–shell architectures and surface modifications, a direction that is expected to fundamentally change how multifunctional IONP platforms are conceptualized and validated [172].

Thus, in the case of IONPs, AI models can also assist in predicting magnetic behavior, biodistribution, and potential toxicity, which are critical parameters for biomedical applications. Additionally, AI-driven approaches can support the design of multifunctional nanoplatforms with improved targeting ligands and surface modifications to enhance tumor specificity and circulation time. The integration of AI with nanotechnology may also contribute to the development of personalized nanomedicine, where patient-specific clinical and genetic data are used to design tailored iron nanoparticle therapies. As computational models and available datasets continue to expand, AI is expected to play an essential role in guiding the rational design of safer and more efficient iron nanoparticle drug delivery systems [175,176].

## 5. Conclusions

Iron-based nanoparticles have emerged as one of the most compelling platforms in nanomedicine, uniquely combining magnetic responsiveness, biodegradability, and versatile surface chemistry within a single, clinically translatable system. This review systematically examined three mechanistically distinct classes of iron-based nanoparticles, iron oxide nanoparticles (IONPs/SPIONs), zero-valent iron nanoparticles (nZVI), and iron-based core–shell architectures, within a unified drug delivery framework, highlighting both their shared properties and their distinct therapeutic profiles.

Among these, SPIONs, particularly magnetite (Fe_3_O_4_) and maghemite (γ-Fe_2_O_3_), remain the most extensively characterized, owing to their superparamagnetic behavior, favorable biocompatibility, and established clinical safety record. Their degradation into endogenous iron metabolic pathways distinguishes them from non-biodegradable nanomaterials. However, as discussed above, this biodegradability is not without risk. Iron ion release can engage Fenton chemistry and generate reactive oxygen species in a dose- and context-dependent manner, particularly in tissues with impaired antioxidant defenses. These considerations must inform dosing strategies and surface engineering choices in future clinical development.

Surface functionalization was identified as a critical determinant of pharmacokinetic performance and therapeutic outcome. PEGylation extends circulation time through immune evasion, while chitosan and dextran coatings offer complementary advantages in drug loading and mucoadhesion. Biomimetic cell membrane coatings, derived from red blood cells, cancer cells, and macrophages, constitute a frontier strategy that bridges nanotechnology and cell biology, conferring homotypic targeting and prolonged circulation via endogenous surface protein repertoires.

Drug loading was reviewed across two principal strategies: non-covalent approaches, including physical adsorption and encapsulation, which offer high loading efficiency but limited release control; and covalent conjugation via stimuli-responsive linkers such as hydrazone and acylhydrazone bonds, which enable pH-triggered, site-specific release in the acidic tumor microenvironment. The therapeutic exploitation of Fenton reactivity through ferroptosis-based cancer therapy represents a particularly promising modality that leverages the intrinsic chemistry of iron nanoparticles, rather than treating it solely as a safety liability.

Despite these advances, significant challenges remain on the path to clinical translation. Batch-to-batch variability in synthesis, long-term biosafety under repeated dosing, limited magnetic targeting efficiency in deep-seated tumors, and the absence of standardized characterization and regulatory frameworks continue to constrain the translation of preclinical promise into clinical outcomes. Addressing these challenges will require coordinated efforts across materials science, pharmacology, clinical medicine, and regulatory science.

Looking ahead, the integration of artificial intelligence in nanoparticle design, enabling predictive optimization of size, surface chemistry, and biodistribution, alongside advances in stimuli-responsive coatings, combination chemo-magnetic-ferroptotic therapies, and precision nanomedicine approaches tailored to patient-specific tumor profiles, collectively define the most promising directions for next-generation iron nanoparticle drug delivery systems. We anticipate that continued progress in these areas will bring iron-based nanoparticles closer to their full clinical potential as safe, targeted, and multifunctional theranostic platforms.

## Figures and Tables

**Table 3:** Comparison of covalent drug conjugation strategies for IONPs.

Nanoparticle System	Drug Loaded	Linker Chemistry	Targeting Ligand	In Vitro Efficacy	Reference
HA-SPION	DOX	Acid-sensitive hydrazone bond	Hyaluronan (HA) → CD44 receptor	IC_50_ = 0.079 μg/mL (SKOV-3); 17.5 μg/mL (NCI/ADR-RES); 4-fold improvement over free DOX in resistant cells; r2* = 431 mM^−1^·s^−1^	[153]
DOX-PEG-SPIO	DOX	pH-sensitive acylhydrazone linkage	None (passive targeting via EPR)	Enhanced cellular uptake and improved antitumor activity vs. free DOX	[154]
Fe_3_O_4_-DOX/SP94	DOX	pH-sensitive hydrazone bond (DOX-dextran); EDC coupling (dextran-NP)	SP94 peptide	Selective accumulation in cancer cells; combined targeting and controlled drug release	[105]

## Data Availability

No new data were created or analyzed in this study. Data sharing is not applicable.

## Abbreviations

The following abbreviations are used in this manuscript:

Abbreviation	Full Term
SPIONs	Superparamagnetic Iron Oxide Nanoparticles
GPX4	Glutathione–glutathione Peroxidase 4
nZVI	Zero-Valent Iron Nanoparticles
IONPs	Iron Oxide Nanoparticles
MNPs	Metal-based Nanoparticles
MRI	Magnetic Resonance Imaging
ROS	Reactive Oxygen Species
PEG	Polyethylene Glycol
DOX	Doxorubicin
RES	Reticuloendothelial System
EPR	Enhanced Permeability and Retention
RNR	Ribonucleotide Reductase
TfR1	Transferrin Receptor 1
IRP	Iron Regulatory Protein
NTBI	Non-Transferrin-Bound Iron
BBB	Blood–Brain Barrier
BTB	Blood-Tumor Barrier
RBC	Red Blood Cell
5-FU	5-Fluorouracil
PLGA	Poly(lactic-co-glycolic acid)
AI	Artificial Intelligence
ML	Machine Learning
PBPK	Physiologically Based Pharmacokinetics
SiRNA	Small Interfering RNA
CRISPR	Clustered Regularly Interspaced Short Palindromic Repeats
AuNPs	Gold Nanoparticles
AgNPs	Silver Nanoparticles
GdNPs	Gadolinium Nanoparticles
uPAR	Urokinase-Type Plasminogen Activator Receptor
APTES	Aminopropyltriethoxysilane
EDC	1-Ethyl-3-(3-dimethylaminopropyl)carbodiimide
HA	Hyaluronan/Hyaluronic Acid

## References

[1] Malamatari M. (2023). The Importance of Drug Delivery in the Clinical Development and Lifecycle of Drug Products with Examples from Authorized Medicinal Products. Processes.

[2] Ebrahimnia M., Alavi S., Vaezi H., Karamat-Iradmousa M., Haeri A. (2024). Exploring the Vast Potentials and Probable Limitations of Novel and Nanostructured Implantable Drug Delivery Systems for Cancer Treatment. EXCLI J..

[3] Rana A., Adhikary M., Singh P.K., Das B.C., Bhatnagar S. (2023). “Smart” drug delivery: A window to future of translational medicine. Front. Chem..

[4] Mehmood S., Iraqui S., Ojha R.K., Sharma N., Marlinda A.R. (2025). Therapeutic Potential and Toxicological Challenges of Metal Nanoparticles in Drug Delivery: A Comprehensive Review. Nanomed. Nanotechnol. Biol. Med..

[5] Shirazi A.N., Vadlapatla R., Koomer A., Nguyen A., Khoury V., Parang K. (2025). Peptide-based inorganic nanoparticles as efficient intracellular delivery systems. Pharmaceutics.

[6] Burlec A.F., Corciova A., Boev M., Batir-Marin D., Mircea C., Cioanca O., Danila G., Danila M., Bucur A.F., Hancianu M. (2023). Current Overview of Metal Nanoparticles’ Synthesis, Characterization, and Biomedical Applications, with a Focus on Silver and Gold Nanoparticles. Pharmaceuticals.

[7] Shubhra Q.T.H. (2023). Iron oxide nanoparticles in magnetic drug targeting and ferroptosis-based cancer therapy. Med. Rev..

[8] Abdelkareem S., El-Sayed M.M.H., Yacoub N., Reda A., Butera V., Camellone M.F., Ritacco I., Shoeib T. (2025). Copper Oxide Nanoparticles as Delivery Vehicles for Different Pt(II)-Drugs: Experimental and Theoretical Evaluation. J. Mater. Chem. B.

[9] Geraldes C.F.G.C. (2024). Manganese Oxide Nanoparticles for MRI-Based Multimodal Imaging and Theranostics. Molecules.

[10] Ravichandran G., Harijan D., Ganapathy N., Prabusankar G., De A., Rengan A.K. (2023). The Multifaceted Role of Degradable Cobalt Nanoparticles: Dual-Target Starvation and Intracellular Acidification Engendering LC3-Associated Whole-Cell Autophagy. ACS Mater. Lett..

[11] Xiao S., Wu S., Shen M., Guo R., Huang Q., Wang S., Shi X. (2009). Polyelectrolyte multilayer-assisted immobilization of zero-valent iron nanoparticles onto polymer nanofibers for potential environmental applications. ACS Appl. Mater. Interfaces.

[12] Chau T.P., Brindhadevi K., Krishnan R., Alyousef M.A., Almoallim H.S., Whangchai N., Pikulkaew S. (2022). A novel synthesis, analysis and evaluation of *Musa coccinea*-based zero-valent iron nanoparticles for antimicrobial and antioxidant activity. Environ. Res..

[13] Laurent S., Boutry S., Mahieu I., Vander Elst L., Muller R.N. (2009). Iron oxide based MR contrast agents: From chemistry to cell labeling. Curr. Med. Chem..

[14] Dulińska-Litewka J., Łazarczyk A., Hałubiec P., Szafrański O., Karnas K., Karewicz A. (2019). Superparamagnetic Iron Oxide Nanoparticles Current and Prospective Medical Applications. Materials.

[15] Smolensky E.D., Park H.Y.E., Zhou Y., Rolla G.A., Marjańska M., Botta M., Pierre V.C. (2013). Scaling laws at the nanosize: The effect of particle size and shape on the magnetism and relaxivity of iron oxide nanoparticle contrast agents. J. Mater. Chem. B.

[16] Wu M., Huang S. (2017). Magnetic nanoparticles in cancer diagnosis, drug delivery and treatment. Mol. Clin. Oncol..

[17] Wu Y., Lu Z., Li Y., Yang J., Zhang X. (2020). Surface modification of iron oxide-based magnetic nanoparticles for cerebral theranostics: Application and prospection. Nanomaterials.

[18] Xie M., Meng F., Wang P., Díaz-García A.M., Parkhats M., Santos-Oliveira R., Cai Y. (2024). Surface engineering of magnetic iron oxide nanoparticles for breast cancer diagnostics and drug delivery. Int. J. Nanomed..

[19] Chen B., Wu W., Wang X. (2011). Magnetic iron oxide nanoparticles for tumor-targeted therapy. Curr. Cancer Drug Targets.

[20] Wang H., Liu Y., Zhang M., Wang J., Wu Z., Li L. (2021). Gadolinium-based nanoparticles for theranostic applications: A review. ACS Appl. Nano Mater..

[21] Shirazi A.N., Vadlapatla R., Koomer A., Zayed H., Marabut P., Parang K. (2026). Gadolinium Nanoparticles: Emerging Platforms Beyond Imaging for Drug Delivery and Theranostics. Pharmaceutics.

[22] Abbasi H., Kouchak M., Mirveis Z., Hajipour F., Khorram M., Rahbar N., Handali S. (2023). What We Need to Know about Liposomes as Drug Nanocarriers: An Updated Review. Adv. Pharm. Bull..

[23] Mir M., Ahmed N., Rehman A.U. (2022). PLGA-Based Nanomedicine: History of Advancement and Development in Clinical Applications of Multiple Diseases. Pharmaceutics.

[24] Al-Thani A.N., Jan A.G., Abbas M., Geetha M., Sadasivuni K.K. (2025). Utilizing Gold Nanoparticles in Plasmonic Photothermal Therapy for Cancer Treatment. Heliyon.

[25] Sarode R.J., Mahajan H.S. (2024). Dendrimers for drug delivery: An overview of its classes, synthesis, and applications. J. Drug Deliv. Sci. Technol..

[26] Li Y., Barmin R.A., Zhang R., Kiessling F., Lammers T., Pallares R.M. (2026). Clinical translation and landscape of superparamagnetic iron oxide nanoparticles. Adv. Drug Deliv. Rev..

[27] Zhao B., Li X., Kong Y., Wang W., Wen T., Zhang Y., Deng Z., Chen Y., Zheng X. (2022). Recent advances in nano-drug delivery systems for synergistic antitumor immunotherapy. Front. Bioeng. Biotechnol..

[28] Khalid-Salako F., Salimi Khaligh S., Fathi F., Demirci O.C., Öncer N., Kurt H., Yüce M. (2025). The Nanocarrier Landscape─Evaluating Key Drug Delivery Vehicles and Their Capabilities: A Translational Perspective. ACS Appl. Mater. Interfaces.

[29] Gupta A.K., Gupta M. (2005). Synthesis and surface engineering of iron oxide nanoparticles for biomedical applications. Biomaterials.

[30] Genc S., Taghizadehghalehjoughi A., Yeni Y., Jafarizad A., Hacimuftuoglu A., Nikitovic D., Docea A.O., Mezhuev Y., Tsatsakis A. (2023). Fe3O4 nanoparticles in combination with 5-FU exert antitumor effects superior to those of the active drug in a colon cancer cell model. Pharmaceutics.

[31] Premanathan M., Karthikeyan K., Jeyasubramanian K., Manivannan G. (2011). Selective toxicity of ZnO nanoparticles toward Gram-positive bacteria and cancer cells by apoptosis through lipid peroxidation. Nanomedicine.

[32] Nasrolahi Shirazi A., Mandal D., Sajid M.I., Stickley D., Nagasawa S., Long J., Parang K., Tiwari R.K. (2021). Cyclic peptide-gadolinium nanocomplexes as siRNA delivery tools. Pharmaceuticals.

[33] Nasrolahi Shirazi A., Park S.E., Rad S., Baloyan L., Mandal D., Sajid M.I., Hall R., Lohan S., Zoghebi K., Parang K. (2020). Cyclic peptide-gadolinium particles for enhanced intracellular delivery. Pharmaceutics.

[34] Nasrolahi Shirazi A., Tiwari R.K., Oh D., Sullivan B., Kumar A., Beni Y., Parang K. (2014). Cyclic peptide-selenium nanoparticles as drug transporters. Mol. Pharm..

[35] Nasrolahi Shirazi A., Neira K., Howlett N., Parang K. (2014). Cyclic peptide-capped gold nanoparticles for enhanced siRNA delivery. Molecules.

[36] Nasrolahi Shirazi A., Tiwari R.K., Oh D., Sullivan B., McCaffrey K., Mandal D., Parang K. (2013). Surface decorated gold nanoparticles by linear and cyclic peptides as molecular transporters. Mol. Pharm..

[37] Nasrolahi Shirazi A., Mandal D., Tiwari R.K., Guo L., Lu W., Parang K. (2013). Cyclic peptide-capped gold nanoparticles as drug delivery systems. Mol. Pharm..

[38] Doan L., Nguyen L.T., Nguyen N.T. (2023). Modifying superparamagnetic iron oxides nanoparticles for doxorubicin delivery carriers: A review. J. Nanopart. Res..

[39] Chen C., Ge J., Gao Y., Chen L., Cui J., Zeng J., Gao M. (2022). Ultrasmall superparamagnetic iron oxide nanoparticles: A next generation contrast agent for magnetic resonance imaging. WIREs Nanomed. Nanobiotechnol..

[40] Graham W., Torbett-Dougherty M., Islam A., Soleimani S., Bruce-Tagoe T.A., Johnson J.A. (2025). Magnetic nanoparticles and drug delivery systems for anti-cancer applications: A review. Nanomaterials.

[41] Mishra S., Yadav M.D. (2024). Magnetic nanoparticles: A comprehensive review from synthesis to biomedical frontiers. Langmuir.

[42] Lapusan R., Borlan R., Focsan M. (2024). Advancing MRI with Magnetic Nanoparticles: A Comprehensive Review of Translational Research and Clinical Trials. Nanoscale Adv..

[43] Camaschella C. (2015). Iron deficiency. N. Engl. J. Med..

[44] Abbaspour N., Hurrell R., Kelishadi R. (2014). Review on iron and its importance for human health. J. Res. Med. Sci..

[45] Rouault T.A. (2013). Iron metabolism in the CNS: Implications for neurodegenerative diseases. Nat. Rev. Neurosci..

[46] Anderson G.J., Frazer D.M. (2017). Current understanding of iron homeostasis. Am. J. Clin. Nutr..

[47] Ganz T., Nemeth E. (2012). Hepcidin and iron homeostasis. Biochim. Biophys. Acta.

[48] Abe C., Miyazawa T., Miyazawa T. (2022). Current Use of Fenton Reaction in Drugs and Food. Molecules.

[49] Ramey G., Deschemin J.C., Durel B., Canonne-Hergaux F., Nicolas G., Vaulont S. (2010). Hepcidin targets ferroportin for degradation in hepatocytes. Haematologica.

[50] Zhong M., Wang Y., Min J., Wang F. (2025). Iron metabolism and ferroptosis in human health and disease. BMC Biol..

[51] Koleini N., Shapiro J.S., Geier J., Ardehali H. (2021). Ironing out mechanisms of iron homeostasis and disorders of iron deficiency. J. Clin. Investig..

[52] Haas J.D., Brownlie T. (2001). Iron deficiency and reduced work capacity. J. Nutr..

[53] Read A.D., Bentley R.E., Archer S.L., Dunham-Snary K.J. (2021). Mitochondrial iron-sulfur clusters: Structure, function, and an emerging role in vascular biology. Redox Biol..

[54] Haber F., Willstätter R. (1931). Unpaarigkeit Und Radikalketten Im Reaktionsmechanismus Organischer Und Enzymatischer Vorgänge. Ber. Dtsch. Chem. Ges..

[55] Haber F., Weiss J., Pope W.J. (1934). The Catalytic Decomposition of Hydrogen Peroxide by Iron Salts. Proc. R. Soc. London. Ser. A Math. Phys. Sci..

[56] Fenton H.J.H. (1894). LXXIII. Oxidation of Tartaric Acid in Presence of Iron. J. Chem. Soc. Trans..

[57] Stanbury D.M. (2022). The Principle of Detailed Balancing, the Iron-Catalyzed Disproportionation of Hydrogen Peroxide, and the Fenton Reaction. Dalton Trans..

[58] Kremer M.L. (2003). The Fenton Reaction. Dependence of the Rate on PH. J. Phys. Chem. A.

[59] Lu H.-F., Chen H.-F., Kao C.-L., Chao I., Chen H.-Y. (2018). A Computational Study of the Fenton Reaction in Different PH Ranges. Phys. Chem. Chem. Phys..

[60] Chen H.-Y. (2019). Why the Reactive Oxygen Species of the Fenton Reaction Switches from Oxoiron(IV) Species to Hydroxyl Radical in Phosphate Buffer Solutions? A Computational Rationale. ACS Omega.

[61] Kastanek F., Spacilova M., Krystynik P., Dlaskova M., Solcova O. (2023). Fenton Reaction–Unique but Still Mysterious. Processes.

[62] Que L. (2007). The road to non-heme oxoferryls and beyond. Acc. Chem. Res..

[63] Oppenheimer S.J. (2001). Iron and its relation to immunity and infectious disease. J. Nutr..

[64] Nemeth E., Ganz T. (2014). Anemia of inflammation. Hematol. Oncol. Clin. N. Am..

[65] Jin X., Tang J., Qiu X., Nie X., Ou S., Wu G., Zhang R., Zhu J. (2024). Ferroptosis: Emerging mechanisms, biological function, and therapeutic potential in cancer and inflammation. Cell Death Discov..

[66] Abdukarimov N., Kokabi K., Kunz J. (2025). Ferroptosis and iron homeostasis: Molecular mechanisms and neurodegenerative disease implications. Antioxidants.

[67] Lozoff B., Georgieff M.K. (2006). Iron deficiency and brain development. Semin. Pediatr. Neurol..

[68] Ward R.J., Zucca F.A., Duyn J.H., Crichton R.R., Zecca L. (2014). The role of iron in brain ageing and neurodegenerative disorders. Lancet Neurol..

[69] Zimmermann M.B., Köhrle J. (2002). The impact of iron deficiency on thyroid metabolism. Thyroid.

[70] Anker S.D., Comin Colet J., Filippatos G., Willenheimer R., Dickstein K., Drexler H., Lüscher T.F., Bart B., Banasiak W., Niegowska J. (2009). Ferric carboxymaltose in patients with heart failure and iron deficiency. N. Engl. J. Med..

[71] Pietrangelo A. (2004). Hereditary hemochromatosis. N. Engl. J. Med..

[72] Sumneang N., Siri-Angkul N., Kumfu S., Chattipakorn S.C., Chattipakorn N. (2020). The effects of iron overload on mitochondrial function, mitochondrial dynamics, and ferroptosis in cardiomyocytes. Arch. Biochem. Biophys..

[73] Georgieff M.K. (2020). Iron deficiency in pregnancy. Am. J. Obstet. Gynecol..

[74] Stockwell B.R., Jiang X., Gu W. (2020). Emerging mechanisms and disease relevance of ferroptosis. Cell.

[75] Ye H., Rouault T.A. (2014). Essential functions of iron-requiring proteins in DNA replication, repair and cell cycle control. Protein Cell.

[76] Sanvisens N., Bañó M.C., Huang M., Puig S. (2011). Regulation of Ribonucleotide Reductase in Response to Iron Deficiency. Mol. Cell.

[77] Petronek M.S., Allen B.G. (2023). Maintenance of genome integrity by the late-acting cytoplasmic iron-sulfur assembly (CIA) complex. Front. Genet..

[78] Torti S.V., Torti F.M. (2021). Iron: The cancer connection. Prog. Mol. Biol. Transl. Sci..

[79] Drakesmith H., Prentice A. (2022). New iron metabolic pathways and chelation targeting strategies affecting the treatment of all types and stages of cancer. Int. J. Mol. Sci..

[80] Torti S.V., Torti F.M. (2013). Iron and cancer: More ore to be mined. Nat. Rev. Cancer.

[81] Dixon S.J., Lemberg K.M., Lamprecht M.R., Skouta R., Zaitsev E.M., Gleason C.E., Patel D.N., Bauer A.J., Cantley A.M., Yang W.S. (2012). Ferroptosis: An iron-dependent form of nonapoptotic cell death. Cell.

[82] Kawabata H. (2022). Iron-induced oxidative stress in human diseases. Cells.

[83] Brandow M.C., Lefebvre C. (2024). Iron overload and toxicity. StatPearls [Internet].

[84] Arbab A.S., Wilson L.B., Ashari P., Jordan E.K., Lewis B.K., Frank J.A. (2005). A model of lysosomal metabolism of dextran coated superparamagnetic iron oxide (SPIO) nanoparticles: Implications for cellular magnetic resonance imaging. NMR Biomed..

[85] Gu J., Xu H., Han Y., Dai W., Hao W., Wang C., Gu N., Xu H., Cao J. (2011). The internalization pathway, metabolic fate and biological effect of superparamagnetic iron oxide nanoparticles in the macrophage-like RAW264.7 cell. Sci. China Life Sci..

[86] De Domenico I., Vaughn M.B., Li L., Bagley D., Musci G., Ward D.M., Kaplan J. (2006). Ferroportin-mediated mobilization of ferritin iron precedes ferritin degradation by the proteasome. EMBO J..

[87] Kowdley K.V., Gochanour E.M., Sundaram V., Shah R.A., Handa P. (2021). Hepcidin signaling in health and disease: Ironing out the details. Hepatol. Commun..

[88] Liu Y., Wang J. (2013). Effects of DMSA-coated Fe3O4 nanoparticles on the transcription of genes related to iron and osmosis homeostasis. Toxicol. Sci..

[89] Wang N., Zhou D., Xu K., Kou D., Chen C., Li C., Ge J., Chen L., Zeng J., Gao M. (2025). Iron homeostasis-regulated adaptive metabolism of PEGylated ultrasmall iron oxide nanoparticles. ACS Nano.

[90] Valdiglesias V., Fernández-Bertólez N., Kiliç G., Costa C., Costa S., Fraga S., Bessa M.J., Pásaro E., Teixeira J.P., Laffon B. (2016). Are iron oxide nanoparticles safe? Current knowledge and future perspectives. J. Trace Elem. Med. Biol..

[91] Laurent S., Forge D., Port M., Roch A., Robic C., Vander Elst L., Muller R.N. (2008). Magnetic iron oxide nanoparticles: Synthesis, stabilization, vectorization, physicochemical characterizations, and biological applications. Chem. Rev..

[92] Pankhurst Q.A., Thanh N.K.T., Jones S.K., Dobson J. (2009). Progress in applications of magnetic nanoparticles in biomedicine. J. Phys. D Appl. Phys..

[93] Wu W., Wu Z., Yu T., Jiang C., Kim W.S. (2015). Recent progress on magnetic iron oxide nanoparticles: Synthesis, surface functional strategies and biomedical applications. Sci. Technol. Adv. Mater..

[94] Jacinto C., Javed Y., Lavorato G., Tarraga W.A., Conde B.I.C., Orozco J.M., Picco A.S., Garcia J., Dias C.S.B., Malik S. (2025). Biotransformation and Biological Fate of Magnetic Iron Oxide Nanoparticles for Biomedical Research and Clinical Applications. Nanoscale Adv..

[95] Wang Y.X. (2015). Current Status of Superparamagnetic Iron Oxide Contrast Agents for Liver Magnetic Resonance Imaging. World J. Gastroenterol..

[96] Baldea I., Iacoviță C., Gurgu R.A., Vizitiu A.S., Râzniceanu V., Mitrea D.R. (2025). Magnetic hyperthermia with iron oxide nanoparticles: From toxicity challenges to cancer applications. Nanomaterials.

[97] Ko M.J., Min S., Hong H., Yoo W., Joo J., Zhang Y.S., Kang H., Kim D.H. (2023). Magnetic Nanoparticles for Ferroptosis Cancer Therapy with Diagnostic Imaging. Bioact. Mater..

[98] Chen Y., Hou S. (2023). Recent progress in the effect of magnetic iron oxide nanoparticles on cells and extracellular vesicles. Cell Death Discov..

[99] Ghazi R., Ibrahim T.K., Abdul Nasir J., Gai S., Ali G., Boukhris I., Rehman Z. (2025). Iron oxide based magnetic nanoparticles for hyperthermia, MRI and drug delivery applications: A review. RSC Adv..

[100] Salehirozveh M., Dehghani P., Mijakovic I. (2024). Synthesis, functionalization, and biomedical applications of iron oxide nanoparticles (IONPs). J. Funct. Biomater..

[101] Mahmoudi M., Hofmann H., Rothen-Rutishauser B., Petri-Fink A. (2012). Assessing the in vitro and in vivo toxicity of superparamagnetic iron oxide nanoparticles. Chem. Rev..

[102] Oberländer J., Speth K.R., Kaltbeitzel A., Lieberwirth I., Landfester K., Mailänder V. (2025). Protein corona fingerprinting of exocytosed nanoparticles reveals time-dependence of exocytosis pathways. Acta Biomater..

[103] Arruebo M., Fernández-Pacheco R., Ibarra M.R., Santamaría J. (2007). Magnetic nanoparticles for drug delivery. Nano Today.

[104] Alexiou C., Schmid R.J., Jurgons R., Kremer M., Wanner G., Bergemann C., Huenges E., Nawroth T., Arnold W., Parak F.G. (2006). Targeting cancer cells: Magnetic nanoparticles as drug carriers. Eur. Biophys. J..

[105] Jin Y., Cheng Z., Yuan Z., Du Y., Tian J., Shao B. (2024). Glucose-regulated protein 78 targeting ICG and DOX loaded hollow Fe3O4 nanoparticles for hepatocellular carcinoma diagnosis and therapy. Int. J. Nanomed..

[106] Mohapatra A., Mohanty A., Park I.-K. (2024). Inorganic Nanomedicine Mediated Ferroptosis: A Synergistic Approach to Combined Cancer Therapies and Immunotherapy. Cancers.

[107] Yang J., Xiong W., Huang L., Li Z., Fan Q., Hu F., Duan X., Fan J., Li B., Feng J. (2024). A mesoporous superparamagnetic iron oxide nanoparticle as a generic drug delivery system for tumor ferroptosis therapy. J. Nanobiotechnol..

[108] Ali A., Zafar H., Zia M., Ul Haq I., Phull A.R., Ali J.S., Hussain A. (2016). Synthesis, Characterization, Applications, and Challenges of Iron Oxide Nanoparticles. Nanotechnol. Sci. Appl..

[109] Jia Y., Yuan M., Yuan H., Huang X., Sui X., Cui X., Tang F., Peng J., Chen J., Lu S. (2012). Co-encapsulation of magnetic Fe_3_O_4_ nanoparticles and doxorubicin into biodegradable PLGA nanocarriers for intratumoral drug delivery. Int. J. Nanomed..

[110] Dadfar S.M., Roemhild K., Drude N.I., von Stillfried S., Knüchel R., Kiessling F., Lammers T. (2019). Iron oxide nanoparticles: Diagnostic, therapeutic and theranostic applications. Adv. Drug Deliv. Rev..

[111] Wu Y.-N., Zhou M., Li S., Li Z., Li J., Wu B., Li G., Li F., Guan X. (2014). Magnetic metal-organic frameworks: γ-Fe2O3@MOFs via confined in situ pyrolysis method for drug delivery. Small.

[112] Vangijzegem T., Lecomte V., Ternad I., Van Leuven L., Muller R.N., Stanicki D., Laurent S. (2023). Superparamagnetic iron oxide nanoparticles (SPION): From fundamentals to state-of-the-art innovative applications for cancer therapy. Pharmaceutics.

[113] Vasić K., Knez Ž, Leitgeb M. (2024). Multifunctional Iron Oxide Nanoparticles as Promising Magnetic Biomaterials in Drug Delivery: A Review. J. Funct. Biomater..

[114] Albukhaty S., Sulaiman G.M., Al-Karagoly H., Mohammed H.A., Hassan A.S., Alshammari A.A.A., Ahmad A.M., Madhi R., Almalki F.A., Khashan K.S. (2024). Iron oxide nanoparticles: The versatility of magnetic and functionalized nanomaterials in targeted drug and gene delivery with effectual magnetofection. J. Drug Deliv. Sci. Technol..

[115] Shapiro B., Kulkarni S., Nacev A., Muro S., Stepanov P.Y., Weinberg I.N. (2015). Open Challenges in Magnetic Drug Targeting. WIREs Nanomed. Nanobiotechnol..

[116] Manescu Paltanea V., Antoniac I., Paltanea G., Nemoianu I.V., Mohan A.G., Antoniac A., Rau J.V., Laptoiu S.A., Mihai P., Gavrila H. (2024). Magnetic hyperthermia in glioblastoma multiforme treatment. Int. J. Mol. Sci..

[117] Armenia I., Cuestas Ayllón C., Torres Herrero B., Bussolari F., Alfranca G., Grazú V., Martínez de la Fuente J. (2022). Photonic and magnetic materials for on-demand local drug delivery. Adv. Drug Deliv. Rev..

[118] Lammers T., Kiessling F., Hennink W.E., Storm G. (2024). Nanomedicine Tumor Targeting. Adv. Mater..

[119] Blanco-Andujar C., Walter A., Cotin G., Bordeianu C., Mertz D., Felder-Flesch D., Begin-Colin S. (2016). Design of iron oxide-based nanoparticles for MRI and magnetic hyperthermia. Nanomedicine.

[120] Wu L., Wen W., Wang X., Huang D., Cao J., Qi X., Shen S. (2022). Ultrasmall iron oxide nanoparticles cause significant toxicity by specifically inducing acute oxidative stress to multiple organs. Part. Fibre Toxicol..

[121] Ansari M.O., Parveen N., Ahmad M.F., Wani A.L., Afrin S., Rahman Y., Jameel S., Khan Y.A., Siddique H.R., Tabish M. (2019). Evaluation of DNA interaction, genotoxicity and oxidative stress induced by iron oxide nanoparticles both in vitro and in vivo: Attenuation by thymoquinone. Sci. Rep..

[122] Wang L., Chen X., Yan C. (2022). Ferroptosis: An emerging therapeutic opportunity for cancer. Genes Dis..

[123] Sun L., Liu H., Ye Y., Lei Y., Islam R., Tan S., Tong R., Miao Y.-B., Cai L. (2023). Smart Nanoparticles for Cancer Therapy. Signal Transduct. Target. Ther..

[124] Li J., Lin C., Zhu Y., Shao C., Wang T., Chen B. (2023). Colorectal cancer cell membrane biomimetic ferroferric oxide nanomaterials for homologous bio-imaging and chemotherapy application. Med. Oncol..

[125] Liu J., Li X., Chen J., Zhang X., Guo J., Gu J., Mei C., Xiao Y., Peng C., Liu J. (2023). Arsenic-loaded biomimetic iron oxide nanoparticles for enhanced ferroptosis-inducing therapy of hepatocellular carcinoma. ACS Appl. Mater. Interfaces.

[126] Hemben A., Chianella I., Leighton G.J.T. (2021). Surface engineered iron oxide nanoparticles generated by inert gas condensation for biomedical applications. Bioengineering.

[127] Siminzar P., Omidi Y., Golchin A., Aghanejad A., Barar J. (2020). Targeted delivery of doxorubicin by magnetic mesoporous silica nanoparticles armed with mucin-1 aptamer. J. Drug Target..

[128] Sagir T., Huysal M., Senel M., Isık S., Burgucu N., Tabakoglu O., Zaim M. (2022). Folic acid conjugated PAMAM-modified mesoporous silica-coated superparamagnetic iron oxide nanoparticles for potential cancer therapy. J. Colloid Interface Sci..

[129] Pasinszki T., Krebsz M. (2020). Synthesis and application of zero-valent iron nanoparticles in water treatment, environmental remediation, catalysis, and their biological effects. Nanomaterials.

[130] Wu Y.N., Yang L.X., Wang P.W., Braet F., Shieh D.B. (2022). From microenvironment remediation to novel anti-cancer strategy: The emergence of zero valent iron nanoparticles. Pharmaceutics.

[131] Zhang C., Bu W., Ni D., Zhang S., Li Q., Yao Z., Zhang J., Yao H., Wang Z., Shi J. (2016). Synthesis of Iron Nanometallic Glasses and Their Application in Cancer Therapy by a Localized Fenton Reaction. Angew. Chem. Int. Ed. Engl..

[132] Anbouhi T.S., Esfidvajani E.M., Nemati F., Haghighat S., Sari S., Attar F., Pakaghideh A., Sohrabi M.J., Mousavi S.E., Falahati M. (2019). Albumin binding, anticancer and antibacterial properties of synthesized zero valent iron nanoparticles. Int. J. Nanomed..

[133] Hsieh C.-H., Hsieh H.-C., Shih F.-S., Wang P.-W., Yang L.-X., Shieh D.-B., Wang Y.-C. (2021). An innovative NRF2 nano-modulator induces lung cancer ferroptosis and elicits an immunostimulatory tumor microenvironment. Theranostics.

[134] Hashemi Z., Ebrahimzadeh M.A., Biparva P., Mortazavi-Derazkola S., Goli H.R., Sadeghian F., Kardan M., Rafiei A. (2020). Biogenic silver and zero-valent iron nanoparticles by Feijoa: Biosynthesis, characterization, cytotoxic, antibacterial and antioxidant activities. Anticancer Agents Med. Chem..

[135] Huang K.-J., Wei Y.-H., Chiu Y.-C., Wu S.-R., Shieh D.-B. (2019). Assessment of zero-valent iron-based nanotherapeutics for ferroptosis induction and resensitization strategy in cancer cells. Biomater. Sci..

[136] Sharma A., Goyal A.K., Rath G. (2018). Development and characterization of gastroretentive high-density pellets lodged with zero valent iron nanoparticles. J. Pharm. Sci..

[137] Salikhov S.V., Ivanenkov Y.A., Krechetov S.P., Veselov M.S., Sviridenkova N.V., Savchenko A.G., Klyachko N.L., Golovin Y.I., Chufarova N.V., Beloglazkina E.K. (2015). Recent advances in the synthesis of Fe3O4@Au core/shell nanoparticles. J. Magn. Magn. Mater..

[138] Hakimian F., Haghiralsadat B.F., Hadian-Ghazvini S., Azizi M., Ghourchian H. (2023). Fe_3_O_4_/Au/porous Au nanohybrid for efficient delivery of doxorubicin as a model drug. Microchim. Acta.

[139] Fang W., Zhu W., Chen H., Zhang H., Hong S., Wei W., Zhao T. (2020). MRI enhancement and tumor targeted drug delivery using Zn2+-doped Fe3O4 core/mesoporous silica shell nanocomposites. ACS Appl. Bio Mater..

[140] Nordin A.H., Ahmad Z., Husna S.M.N., Ilyas R.A., Azemi A.K., Ismail N., Nordin M.L., Ngadi N., Siti N.H., Nabgan W. (2023). The State of the Art of Natural Polymer Functionalized Fe3O4 Magnetic Nanoparticle Composites for Drug Delivery Applications: A Review. Gels.

[141] Ebadi M., Rifqi Md Zain A., Tengku Abdul Aziz T.H., Mohammadi H., Tee C.A.T., Rahimi Yusop M. (2023). Formulation and Characterization of Fe3O4@PEG Nanoparticles Loaded Sorafenib; Molecular Studies and Evaluation of Cytotoxicity in Liver Cancer Cell Lines. Polymers.

[142] Jain T.K., Morales M.A., Sahoo S.K., Leslie-Pelecky D.L., Labhasetwar V. (2005). Iron oxide nanoparticles for sustained delivery of anticancer agents. Mol. Pharm..

[143] Yallapu M.M., Foy S.P., Jain T.K., Labhasetwar V. (2010). PEG-functionalized magnetic nanoparticles for drug delivery and magnetic resonance imaging applications. Pharm. Res..

[144] Ozkahraman B., Storozhuk L., Guo D., Tung L.D., Mourdikoudis S., Thanh N.T.K. (2026). Smart pH-responsive magnetic iron oxide nanoflower-chitosan nanogels for controlled drug delivery in cancer therapy. Nanoscale.

[145] Mura S., Nicolas J., Couvreur P. (2013). Stimuli-responsive nanocarriers for drug delivery. Nat. Mater..

[146] Kim J., Kim H.S., Lee N., Kim T., Kim H., Yu T., Song I.C., Moon W.K., Hyeon T. (2008). Multifunctional uniform nanoparticles composed of a magnetite nanocrystal core and a mesoporous silica shell for magnetic resonance and fluorescence imaging and for drug delivery. Angew. Chem. Int. Ed..

[147] Gautier J., Allard-Vannier E., Burlaud-Gaillard J., Domenech J., Chourpa I. (2015). Efficacy and hemotoxicity of stealth doxorubicin-loaded magnetic nanovectors on breast cancer xenografts. J. Biomed. Nanotechnol..

[148] El-Boubbou K., Ali R., Bahhari H.M., AlSaad K.O., Nehdi A., Boudjelal M., AlKushi A. (2016). Magnetic fluorescent nanoformulation for intracellular drug delivery to human breast cancer, primary tumors, and tumor biopsies: Beyond targeting expectations. Bioconjug. Chem..

[149] Yang L., Cao Z., Sajja H.K., Mao H., Wang L., Geng H., Xu H., Jiang T., Wood W.C., Nie S. (2008). Development of receptor targeted magnetic iron oxide nanoparticles for efficient drug delivery and tumor imaging. J. Biomed. Nanotechnol..

[150] Peng M., Li H., Luo Z., Kong J., Wan Y., Zheng L., Zhang Q., Niu H., Vermorken A., Van de Ven W. (2015). Dextran-coated superparamagnetic nanoparticles as potential cancer drug carriers in vivo. Nanoscale.

[151] Singh A., Bajpai J., Bajpai A.K., Mongre R.K., Lee M.-S. (2020). Encapsulation of cytarabine into casein coated iron oxide nanoparticles (CCIONPs) and study of in vitro drug release and anticancer activities. J. Drug Deliv. Sci. Technol..

[152] Vasić K., Knez Ž, Leitgeb M. (2026). Iron nanoparticles for drug delivery. Inorganic Biomaterials for Drug Delivery.

[153] El-Dakdouki M.H., Zhu D.C., El-Boubbou K., Kamat M., Chen J., Li W., Huang X. (2012). Development of multifunctional hyaluronan-coated nanoparticles for imaging and drug delivery to cancer cells. Biomacromolecules.

[154] Zhu L., Wang D., Wei X., Zhu X., Li J., Tu C., Su Y., Wu J., Zhu B., Yan D. (2013). Multifunctional pH-sensitive superparamagnetic iron-oxide nanocomposites for targeted drug delivery and MR imaging. J. Control. Release.

[155] Wang Y., Jia H.-Z., Han K., Zhuo R.-X., Zhang X.-Z. (2013). Theranostic magnetic nanoparticles for efficient capture and in situ chemotherapy of circulating tumor cells. J. Mater. Chem. B.

[156] Aram E., Moeni M., Abedizadeh R., Sabour D., Sadeghi-Abandansari H., Gardy J., Hassanpour A. (2022). Smart and multifunctional magnetic nanoparticles for cancer treatment applications: Clinical challenges and future prospects. Nanomaterials.

[157] Tran N., Webster T.J. (2010). Magnetic nanoparticles: Biomedical applications and challenges. J. Mater. Chem..

[158] Unsoy G., Gunduz U. (2017). Targeted drug delivery via chitosan-coated magnetic nanoparticles. Nanostructures for Drug Delivery.

[159] Janowicz P.W., Boele T., Maschmeyer R.T., Gholami Y.H., Kempe E.G., Stringer B.W., Stoner S.P., Zhang M., du Toit-Thompson T., Williams F. (2025). Enhanced detection of glioblastoma vasculature with superparamagnetic iron oxide nanoparticles and MRI. Sci. Rep..

[160] Shaghaghi Z., Mansouri R., Nosrati S., Alvandi M. (2025). Multimodal imaging in cancer detection: The role of SPIONs and USPIONs as contrast agents for MRI, SPECT, and PET. Future Oncol..

[161] Li Y., Chen J., Xia Q., Shang J., He Y., Li Z., Chen Y., Gao F., Yu X., Yuan Z. (2024). Photothermal Fe3O4 nanoparticles induced immunogenic ferroptosis for synergistic colorectal cancer therapy. J. Nanobiotechnol..

[162] Vasić K., Knez Ž, Leitgeb M. (2020). Iron nanoparticles for drug delivery. Int. J. Mol. Sci..

[163] Lim S.H., Yee G.T., Khang D. (2024). Nanoparticle-based combinational strategies for overcoming the blood-brain barrier and blood-tumor barrier. Int. J. Nanomed..

[164] Esmaeilpour D., Sivakumar P.M., Khosravi A., Zarepour A., Taj M.B., Nazarzadeh Zare E., Sillanpää M., Karimi-Maleh H. (2026). Polysaccharide-Functionalized Gold, Silver, and Iron Oxide Nanoparticles for siRNA Delivery: The Role of Artificial Intelligence in Design and Optimization. Materials Today Bio.

[165] Tang J., Sun Q., Xie Y., Zheng Q., Ding Y. (2023). Virus-like Iron-Gold Heterogeneous Nanoparticles for Drug Target Screening. Anal. Chem..

[166] Xie X., Yu T., Li X., Zhang N., Foster L.J., Peng C., Huang W., He G. (2023). Recent advances in targeting the “undruggable” proteins: From drug discovery to clinical trials. Signal Transduct. Target. Ther..

[167] Raoof F., Munawar A., Ahmad M., Rizvi S.F., Ali Z., Shahid A.B. (2023). Multifunctional Iron Oxide Nanocarriers Synthesis for Drug Delivery, Diagnostic Imaging, and Biodistribution Study. Appl. Biochem. Biotechnol..

[168] Estelrich J., Escribano E., Queralt J., Busquets M.A. (2015). Iron oxide nanoparticles for magnetically guided and magnetically responsive drug delivery. Int. J. Mol. Sci..

[169] He M., Dan Y., Chen M., Dong C.M. (2023). Biocompatible polymer-modified nanoplatform for ferroptosis-enhanced combination cancer therapy. Macromol. Biosci..

[170] Zhang Z., Xiang Y., Laforet J., Spasojevic I., Fan P., Heffernan A., Eyler C.E., Wood K.C., Hartman Z.C., Reker D. (2025). TuNa-AI: A Hybrid Kernel Machine To Design Tunable Nanoparticles for Drug Delivery. ACS Nano.

[171] Lin Z., Chou W.C., Cheng Y.H., He C., Monteiro-Riviere N.A., Riviere J.E. (2022). Predicting nanoparticle delivery to tumors using machine learning and artificial intelligence approaches. Int. J. Nanomed..

[172] Nedkyalkova M., Vasighi M., Lattuada M. (2025). Integrating surface chemistry properties and machine learning to map the toxicity landscape of superparamagnetic iron oxide nanoparticles. Chemosphere.

[173] He S., Barón A., Munteanu C.R., de Bilbao B., Casañola-Martin G.M., Chelu M., Musuc A.M., Bediaga H., Ascencio E., Castellanos-Rubio I. (2025). Drug release nanoparticle system design: Data set compilation and machine learning modeling. ACS Appl. Mater. Interfaces.

[174] Tiwari A., Widodo, Krisnawati D.I., Chen C.Y., Kuo T.R. (2026). AI-driven nanomedicine for cancer theranostics. Mol. Cancer.

[175] Paul D., Sanap G., Shenoy S., Kalyane D., Kalia K., Tekade R.K. (2021). Artificial intelligence in drug delivery and nanomedicine. Pharmaceutics.

[176] Basu A., Sarkar A., Maulik U. (2020). Machine learning approaches for nanomaterial design and biomedical applications. ACS Nano.

